# The ecology of the *Drosophila*-yeast mutualism in wineries

**DOI:** 10.1371/journal.pone.0196440

**Published:** 2018-05-16

**Authors:** Allison S. Quan, Michael B. Eisen

**Affiliations:** 1 Department of Molecular and Cell Biology, University of California, Berkeley, California, United States of America; 2 Department of Integrative Biology, University of California, Berkeley, California, United States of America; 3 Howard Hughes Medical Institute, University of California, Berkeley, California, United States of America; University of Strasbourg, FRANCE

## Abstract

The fruit fly, *Drosophila melanogaster*, is preferentially found on fermenting fruits. The yeasts that dominate the microbial communities of these substrates are the primary food source for developing *D*. *melanogaster* larvae, and adult flies manifest a strong olfactory system-mediated attraction for the volatile compounds produced by these yeasts during fermentation. Although most work on this interaction has focused on the standard laboratory yeast *Saccharomyces cerevisiae*, a wide variety of other yeasts naturally ferment fallen fruit. Here we address the open question of whether *D*. *melanogaster* preferentially associates with distinct yeasts in different, closely-related environments. We characterized the spatial and temporal dynamics of *Drosophila*-associated fungi in Northern California wineries that use organic grapes and natural fermentation using high-throughput, short-amplicon sequencing. We found that there is nonrandom structure in the fungal communities that are vectored by flies both between and within vineyards. Within wineries, the fungal communities associated with flies in cellars, fermentation tanks, and pomace piles are distinguished by varying abundances of a small number of yeast species. To investigate the origins of this structure, we assayed *Drosophila* attraction to, oviposition on, larval development in, and longevity when consuming the yeasts that distinguish vineyard microhabitats from each other. We found that wild fly lines did not respond differentially to the yeast species that distinguish winery habitats in habitat specific manner. Instead, this subset of yeast shares traits that make them attractive to and ensure their close association with *Drosophila*.

## Introduction

All animals interact with microbes, and it is increasingly clear that the collection of microbes with which an animal interacts can have a dramatic impact on its physiology, behavior, and other phenotypes [1–5]. Some of the microbes associated with animals in the wild are highly specific and acquired through dedicated mechanisms that ensure the robust maintenance of their interaction [6–8]. Other associations, however, are more contingent, and involve microbes acquired as the animal navigates a microbe rich environment. While this latter class has received less attention, studying the contingent microbiome of wild animals can reveal important details of natural history, ecology, and behavior.

The microbiome of the fruit fly, *Drosophila melanogaster*, represents an interesting mix of obligate and contingent microbial associations [9]. In nature, *D*. *melanogaster* is found on or near fermenting substrates, on which they preferentially oviposit, as *D*. *melanogaster* larvae (and indeed those of most *Drosophila* species) feed on microbes, particularly yeasts [10,11]. Yeasts benefit from visits by adult flies, who vector them from site to site, enabling their dispersal and colonization of new substrates [12,13]. This association is mediated by a strong, olfactory-based attraction of adult *D*. *melanogaster* to the volatile compounds produced during yeast fermentation [14–16].

A growing body of work has investigated the interaction between *D*. *melanogaster* and the brewer’s yeast, *Saccharomyces cerevisiae*, in the laboratory. Adult fruit flies prefer substrates inoculated with yeast over any other sterile substrate [15], and under laboratory conditions, *D*. *melanogaster* can discriminate between and prefers some strains of *S*. *cerevisiae* over others based on volatile profile alone [17–19]. While the interaction between flies and yeasts is clear, the specificity of this interaction in nature has been poorly studied.

Both *D*. *melanogaster* and *S*. *cerevisiae* are found in abundance in wineries, a habitat more natural than a controlled laboratory, but more accessible than a completely wild ecosystem [20]. A variety of non-*Saccharomyces* yeasts are observed during spontaneous fermentation, a winemaking practice in which only the yeasts found on the grapes at the time of harvest, and those introduced naturally or incidentally after harvest, are used for fermentation [21,22]. However, little is understood about the movement of non-*Saccharomyces* yeasts in vineyards, although insects are acknowledged as potentially important vectors [23–27]. Given that drosophilids are closely associated with yeast throughout their entire lifecycle, flies are likely candidates for vineyard and winery yeast dispersal [13,28,29]. However, the yeasts associated with vineyard and winery *Drosophila* have yet to be thoroughly characterized.

In a broader context, investigating the degree of specificity of the fly-yeast mutualism in nature can help reveal both the constraints and plasticity of natural mutualisms. While several studies have characterized the yeasts vectored by *Drosophila* in vineyards and wineries using culture-based methods [20,30], we present here a comprehensive study of the relationship between flies and yeast in wineries, using high-throughput, amplicon sequencing of the fungi associated with flies, coupled with well-established *Drosophila* behavior assays using both the yeast isolates and fly lines isolated from the same wineries. We demonstrate that *Drosophila* vector a distinct set of yeasts in wineries and exhibit a generally positive behavioral response towards commonly vectored yeasts. This suggests that the fly-yeast mutualism is not as specific as laboratory experiments indicate, and that flies interact with a diversity of yeast species in different ecological contexts.

## Results

### *Drosophila* vector wine yeasts in wineries

**Fig 1:**
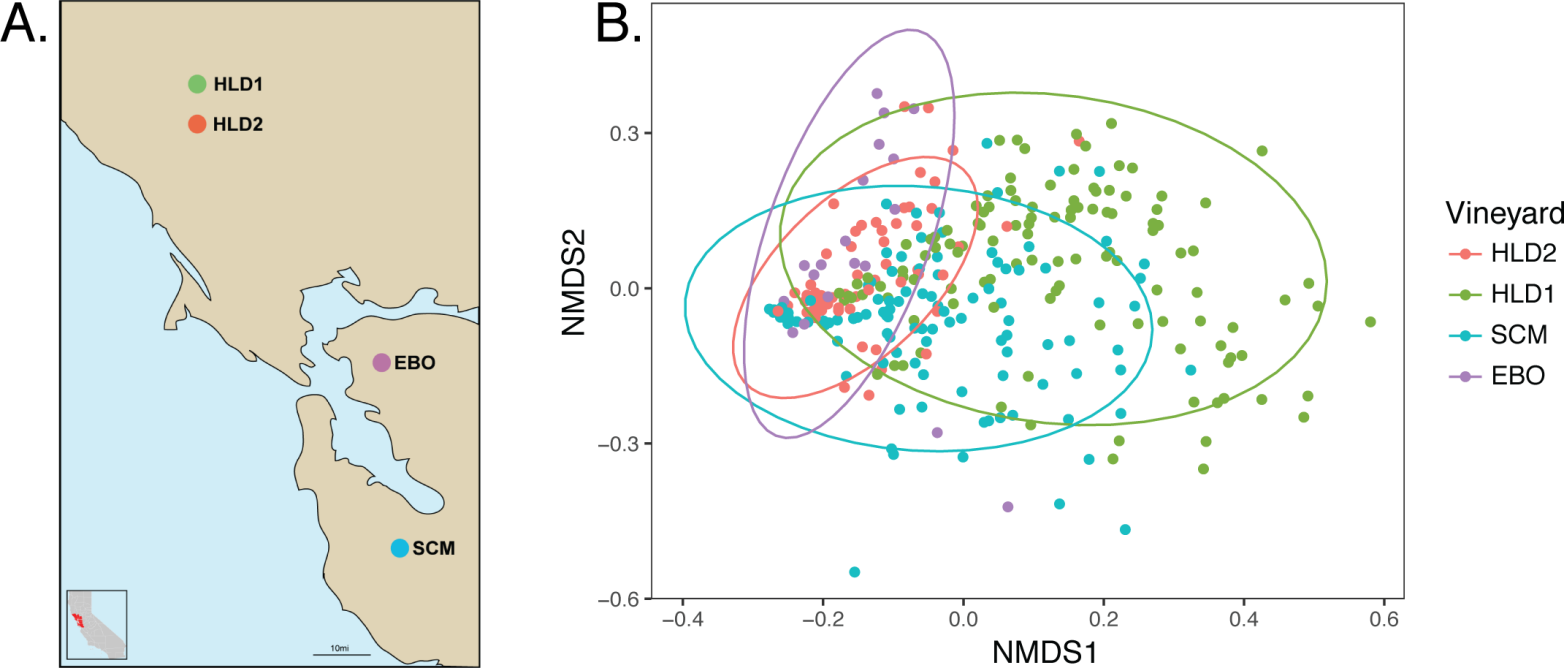
Fungal communities vectored by *Drosophila* are distinct between wineries. (A) Geographic locations of wineries sampled from 2015 and 2016 in the San Francisco Bay Area. (B) Bray-Curtis dissimilarity NMDS of fungal communities vectored by *Drosophila* in wineries (ADONIS: R^2^ = 0.129, *p* = 0.001). Each sample was rarefied to 1000 sequences and is represented by a single point, color-coded by winery. Note, HLD2 and EBO have fewer samples because these wineries were only sampled in 2016.

To identify the fungi vectored by flies, we collected adult *Drosophila* in three areas–fermentation tanks, cellars, and pomace piles–in four different wineries over two harvest seasons in the San Francisco Bay Area, California, USA (Fig 1A). In our initial harvest season, we collected in two wineries, one in Healdsburg, CA (HLD1) and the other in the Santa Cruz Mountains (SCM). We collected adult *Drosophila* every two weeks from May 2015 to November 2015. To expand our study in 2016, we collected flies in a four wineries, HLD1, SCM, HLD2 (also in Healdsburg, CA), and EBO (Orinda, CA) at a single time point from each winery in late September 2016—early October 2016.

DNA from whole, adult flies was extracted and short-amplicon sequencing targeting the universal fungal internal transcribed spacer region (ITS) was performed to characterize fungal community composition [31,32]. After quality filtering and processing, we clustered a total of 7,609,820 fungal ITS reads into 399 operational taxonomic units (OTUs) (S1 Table). When rarefied to 1000 sequences per sample, the overall mean OTU richness per fly associated fungal community for each winery ranged from 19.741 +/- 7.673 to 39.771 +/- 10.537 OTUs (S1 Fig, S2 Table). We found that *Drosophila* species that were not *D*. *melanogaster* or its sister species *D*. *simulans* carried subtly but significantly different fungal communities (R_ANOSIM_ = 0.018, p<0.001, S2 Fig) so we omitted these samples from our subsequent analysis. Removal of these samples resulted in 308 OTUs (S1 Data).

As expected, yeast species dominate the fungal communities vectored by *Drosophila*. The phylum Ascomycota, which includes many yeast species, represented the bulk of the fly-associated fungal taxa (average relative abundance: 95.6%). At the species level, fungal communities were dominated by *Hanseniaspora uvarum* (30.2% average relative abundance across all samples), *Pichia manshurica* (11.5%), *Issatchenkia orientalis* (10%), and members of the genus *Pichia* we could not identify at the species level (4.4%). Although *S*. *cerevisiae* is the dominant yeast in late stage fermentations, it was unevenly represented in the fungal communities vectored by *Drosophila*. Only 8.8% of the samples collected contained *S*. *cerevisiae* reads and of these samples, the relative abundance of *S*. *cerevisiae* ranged from 0.1% to 81.9% with no correlation to any particular winery or winery microhabitat.

In the laboratory, flies exhibit a strong attraction to fermentation volatiles, so it was unsurprising to find that drosophilds were associated with a range of fermentative yeast species commonly found in winery environments. However, the weak association with *S*. *cerevisiae* we observed was noteworthy given that *S*. *cerevisiae* is almost exclusively used in behavior assays investigating the fly-yeast mutualism in the laboratory. As previously suggested by Hoang et al [33], it is possible that other yeast species may have a stronger association with *Drosophila* than *S*. *cerevisiae*. Since we did observe a strong association between flies and non-*Saccharomyces* yeasts, we next asked if these associations were homogeneous across all winery *Drosophila* or dynamic across space and time.

### Nonrandom structure in the fungal communities associated with *Drosophila* between and within wineries

To elucidate the spatial and temporal patterns of the fly-fungi relationship, we asked if fungal community patterns could be distinguished between winery *Drosophila* populations. We found that the fungal communities vectored by flies are not randomly distributed and are significantly different between wineries (Fig 1B, Bray-Curtis R_ANOSIM_ = 0.129, p<0.001). These observations are consistent with what is known about the microbial communities present on wine grapes, which are predominantly defined by regional geography [34,35].

Unsurprisingly, fruit flies are found predominately in areas of active fermentation or containing products of fermentation. To reflect this, we focused our drosophilid collections in three main areas in each winery: fermentation tanks, where primary fermentation occurs; cellars, where wine is aged; and the pomace pile, where grape berry waste is discarded. Adult flies are abundant at all of these winery microhabitats during wine production.

**Fig 2:**
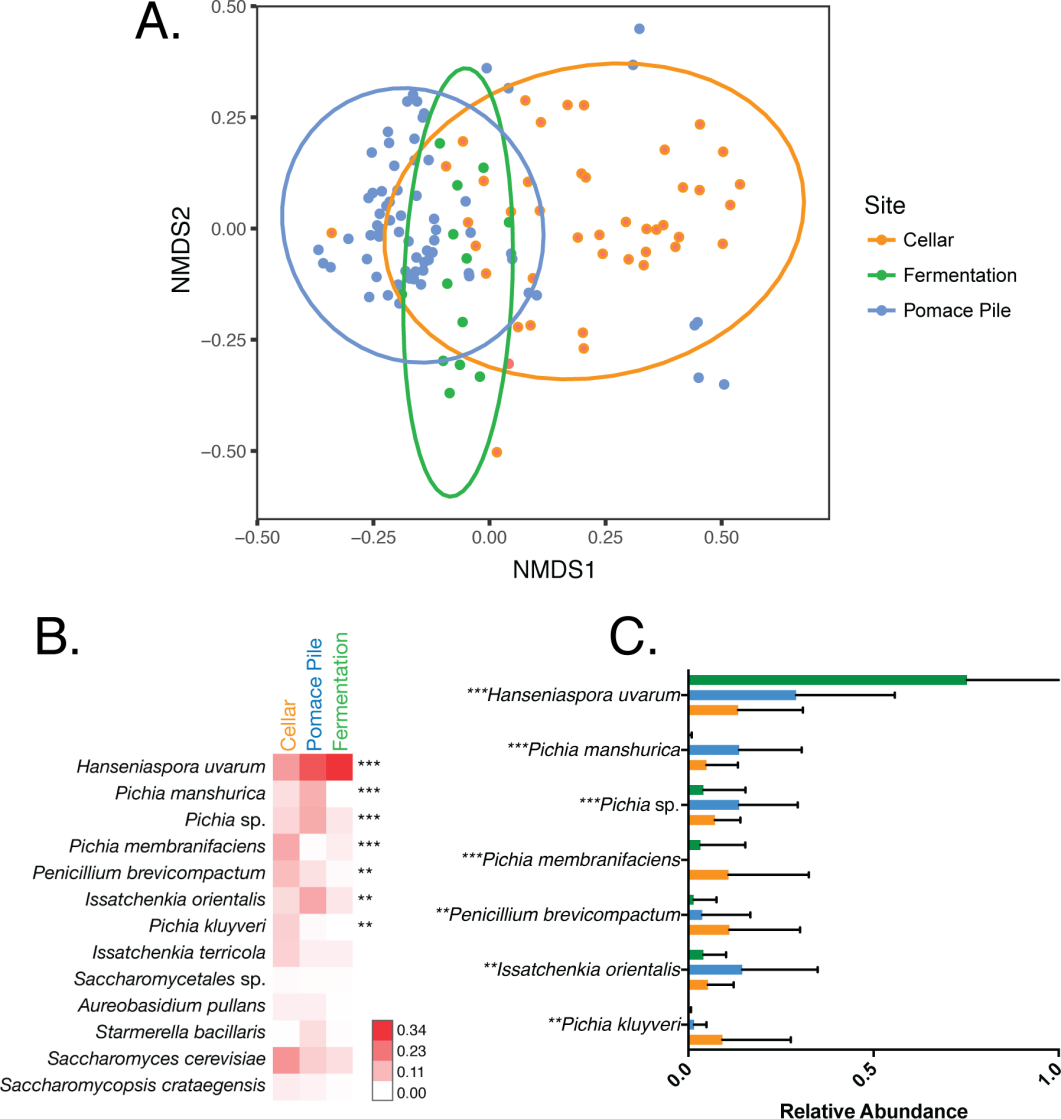
Within wineries, the fungal communities vectored by *Drosophila* are distinct between winery habitat and distinguished by the relative abundances of a few yeast species. (A) Bray-Curtis dissimilarity NMDS of fungal communities vectored by *Drosophila* in HLD1 in 2015 and 2016 (ADONIS: R^2^ = 0.166, *p* = 0.001). Each sample was rarefied to 1000 sequences and is represented by a single point, color-coded by winery area. (B) Heatmap comparing the average relative abundances of all fungal species representing >1% of the total fungal community in each winery area. Each row represents a single fungal species. Stars to the right denote fungal taxa that have significantly different relative abundances between winery areas (one-way ANOVA with Bonferroni error correction, ns: not significant, *: p<0.05, **: p<0.01, ***: p<0.001, ****: p<0.0001.). (C) Bar graphs of the relative abundances of fungal taxa that are significantly different between winery areas.

Within the HLD1 winery, we observed fungal community structure in the fungi vectored by flies between these three winery areas (Fig 2A, Bray-Curtis R_ANOSIM_ = 0.166, p<0.001). A single yeast species, *Hanseniaspora uvarum* (75.3%), dominated the fungal communities vectored by *Drosophila* collected from fermentation tanks. *Saccharomyces cerevisiae* (17.6%), *Hanseniaspora uvarum* (16.1%), *Pichia membranificiens* (14%), and *Penicillium brevicompactum* (10.7%) were overrepresented in the fungal communities carried by flies from the cellars while the pomace pile flies vectored primarily *Hanseniaspora uvarum* (28.2%), *Issachenkia orientalis* (14.6%), and *Pichia* species, such as *Pichia manshurica* (12.9%).

*Drosophila* in different winery areas carried many of the same fungal taxa but the relative abundances of these species distinguished fungal communities in one area from another (Fig 2B and 2C). Of these fungal species, the relative abundances of only six taxa were significantly different between the fungal communities vectored by flies in these specific winery areas: *Hanseniaspora uvarum*, *Pichia manshurica*, *Pichia membranificiens*, *Penicillium brevicompactum*, *Issatchenkia orientalis*, and *Pichia kluyveri* (Fig 2C). All of these yeast species are commonly found in vineyards and wineries [35]. Studies at other wineries have shown that winery equipment and processing surfaces harbor distinct microbial communities that change rapidly over time [34]. While we also observed distinct fly-associated fungal communities in different winery areas, the makeup of these communities do not fluctuate over time and do not completely mirror the previously identified fungal communities colonizing the fermentation tanks and cellar surfaces in other studies. Instead, *Drosophila* carry a subset of these taxa, suggesting that flies might play a role in shaping or maintaining the fungal community composition in these areas and only a subset of the yeasts from these fungal communities have a direct mutualistic relationship with flies.

### *Drosophila melanogaster* do not prefer yeast species representative of the winery area from which they were collected

We next asked how the fungal community structure between different winery areas is established. If flies actively modulate their associated fungal communities, we expected flies to prefer the yeast species characteristic of the fungal communities in the winery area from which they were established. We expected those preferences to manifest themselves in fly behaviors that are closely associated with the presence of yeast, such as olfactory attraction, oviposition, larval development, and longevity.

To test these hypotheses, we quantified the behaviors of four isofemale *Drosophila melanogaster* lines that were established from the three winery areas towards yeast isolates that were cultured and isolated from flies in the winery (Table 1). Founders of the fly and yeast lines were collected from the SCM and HLD1 wineries. We selected a panel of six yeast species that most strongly distinguished each winery area from the others (Fig 2C). Each winery area was represented by a single yeast species except for the pomace pile, which was represented by two because *Pichia manshurica* (isolate P2) was unable to ferment in liquid grape juice. We also included two controls in the yeast panel. *Issachenkia terricola* (yeast isolate CTLns) was included because it was vectored by all flies and did not distinguish one winery area from the others. Finally, *S*. *cerevisiae* (isolate CTLsc) was included as it is most often used in *Drosophila* behavior experiments in the laboratory and is generally attractive to *Drosophila melanogaster*, although we did not find that it was vectored frequently by wild flies in the winery.

**Table 1:** Fly lines and yeast isolates used in the behavior assays.

Isolate/ Fly Line	Type	Species	Vineyard	Winery Habitat	Year Collected	Month Collected
C1	Yeast isolate	*Pichia kluyveri*	HLD1	Cellar	2015	August
F1	Yeast isolate	*Hanseniaspora uvarum*	SCM	Fermentation	2014	October
P1	Yeast isolate	*Issachenkia orientalis*	SCM	Pomace pile	2015	September
P2	Yeast isolate	*Pichia manshurica*	HLD1	Pomace pile	2015	August
CTLsc	Yeast isolate	*Saccharomyces cerevisiae*	SCM	Fermentation	2014	October
CTLns	Yeast isolate	*Issachenkia terricola*	SCM	Fermentation	2015	September
FermA	Isofemale fly line	*Drosophila melanogaster*	SCM	Fermentation	2014	October
FermB	Isofemale fly line	*Drosophila melanogaster*	SCM	Fermentation	2014	October
CellarA	Isofemale fly line	*Drosophila melanogaster*	HLD1	Cellar	2015	August
PPA	Isofemale fly line	*Drosophila melanogaster*	HLD1	Pomace pile	2015	August

#### Olfactory preference

Because *Drosophila* initially rely on olfactory cues to locate yeasts [16,36], we first tested differential attraction for the yeast species that distinguish the fly-associated fungal communities from different winery areas. We used a simple, olfactory-based assay previously developed in our lab [17] to quantify fly preference, and tested pairwise comparisons of a smaller yeast panel, with a single yeast representing each winery area.

**Fig 3:**
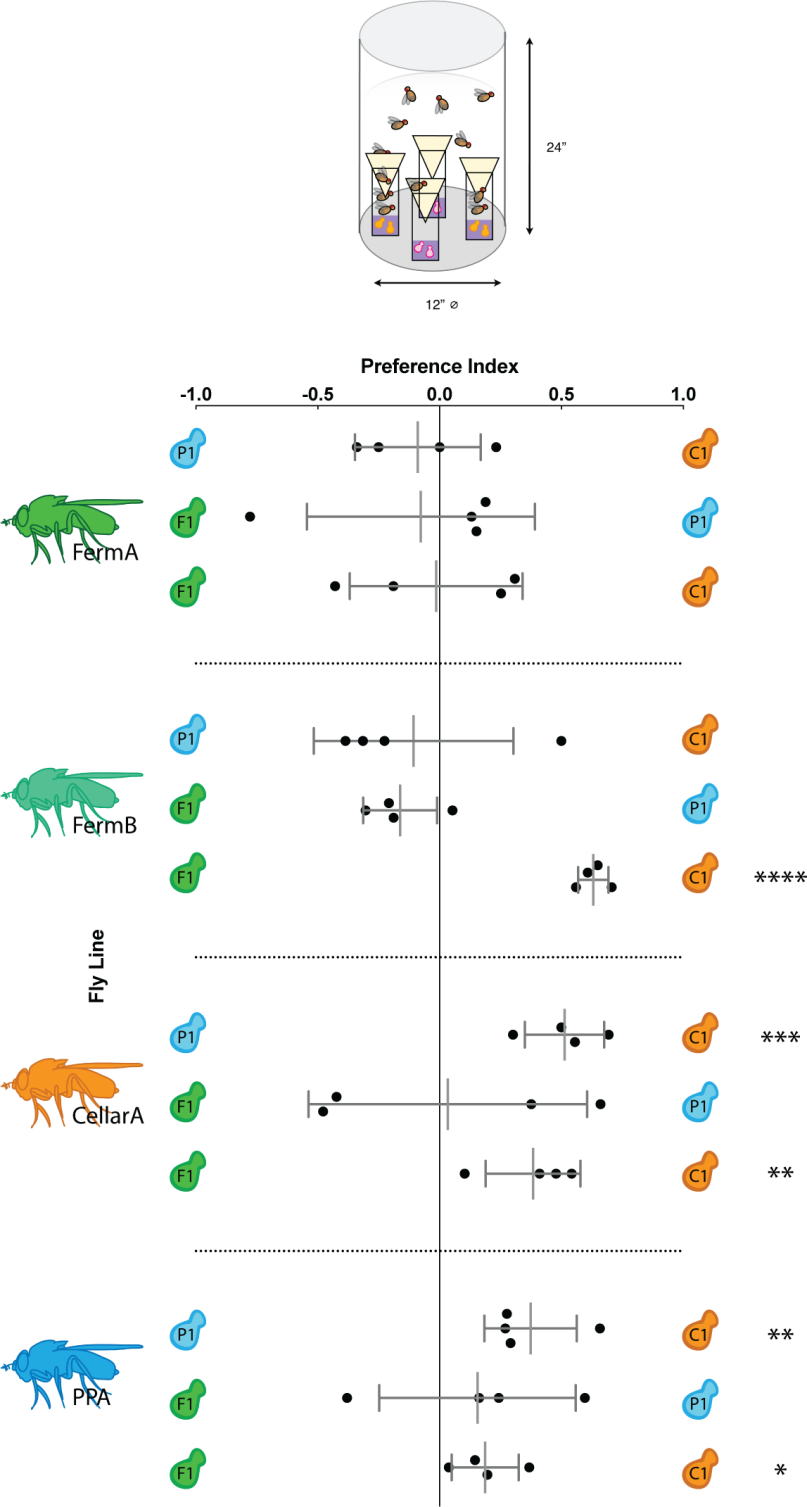
Based on olfactory cues, Drosophila do not prefer the yeast associated with their winery area. *Drosophila* lines tested are denoted by fly icons to the left and yeast species being compared are denoted by yeast symbols on the left and right axes. For a given comparison between yeast species A and B, a positive PI indicates a preference for yeast A, a negative PI indicates a preference for B, and a PI of 0 indicates no preference. Black dots indicate trial replicates. Short grey lines represent standard deviation and longer grey lines represent the mean of all trials. Stars to the left denote significantly different preferences between the two yeast species being tested (multiple t-tests with a Bonferroni correction, *: p<0.05, **: p<0.01, ***: p<0.001, ****: p<0.0001).

Although we did find significant preferences between yeast species, these preferences did not reflect winery area and were variable between fly lines (Fig 3). In all lines except for FermA, *Pichia kluyveri* (yeast isolate C1) was more attractive than both *Hanseniaspora uvarum* (yeast isolate F1) and *Issachenkia orientalis* (yeast isolate P1). Interestingly, FermA was the only fly line that was equally attracted to all three yeast species.

Fly lines did not prefer the yeast species that we had designated as representative of the fungal communities vectored by flies in their winery area. Instead, fly lines were more attracted to some yeasts over others with variability between lines. While the attractiveness of these yeast species varies, these data suggest that the yeast species tested in this study produce metabolites that are generally attractive to *Drosophila*.

#### Oviposition

**Fig 4:**
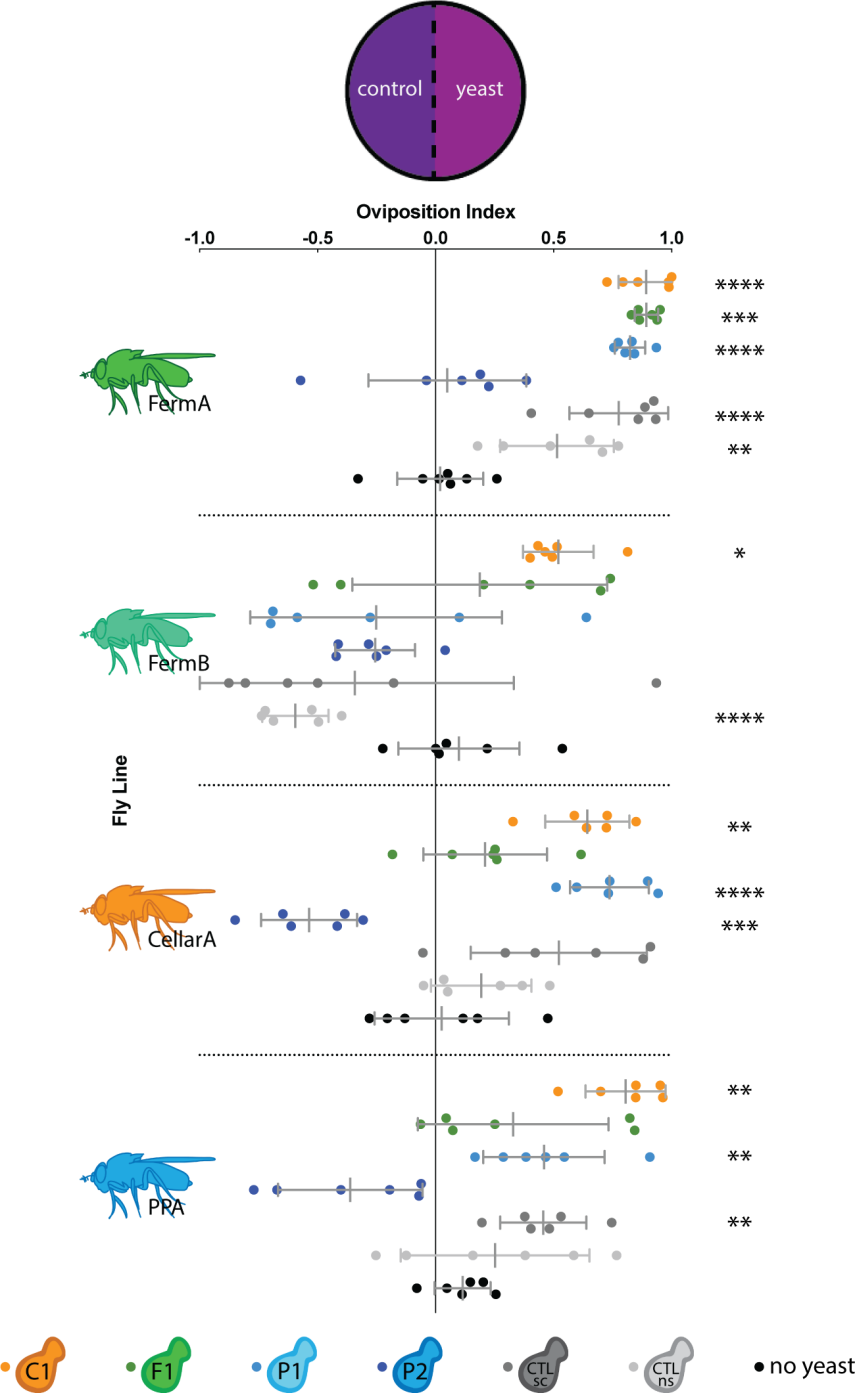
Most fly-associated yeasts elicit a generally positive oviposition response with variability between fly lines. A positive OI indicates an ovipositional preference for the yeast side, a negative OI indicates an ovipositional preference for the control side, and an OI of 0 indicates no preference. *Drosophila* lines tested are denoted by fly icons to the left. Individual replicates are represented by dots and are color-coded by yeast species. Short grey lines represent standard deviation and longer grey lines represent the mean of all trials. Stars to the left denote significantly different oviposition preferences between the two sides (multiple t-tests with a Bonferroni correction, *: p<0.05, **: p<0.01, ***: p<0.001, ****: p<0.0001).

Where female flies choose to lay eggs is strongly coupled with offspring fitness [37,38]. We hypothesized that if *Drosophila* actively modulate the fungal communities they vector, female flies would prefer to oviposit on substrate inoculated with yeast representative of their winery area. To determine if this specificity exists, we tested female oviposition preference for sterile grape substrate or grape substrate inoculated with a single yeast species from our yeast panel (Fig 4).

*Pichia kluyveri* (yeast isolate C1) was the only yeast species on the panel that elicited a significant, positive oviposition response (Fig 4) for all fly lines tested, which is consistent with olfactory preference results. In contrast, *Pichia manshurica* (yeast isolate P2) was the only yeast that elicited no preference or a negative oviposition response in all fly lines. Analysis of the metabolites produced by *Pichia manshurica* using gas-chromotography, mass-spectroscopy (GC-MS) showed that this yeast species is unable to ferment and produce volatile metabolites in liquid grape media (S3 Fig). The lack of volatile attractants may explain why *Pichia manshurica* elicits oviposition responses that mirror those of a sterile media control. Other yeasts in the panel, which fermented successfully, elicited intermediate ovipositional responses. These ovipositional preference patterns are not correlated with winery area. Instead, *Drosophila* seem to follow a general behavioral trend in response to the yeast panel, where some yeasts are more or less desirable oviposition substrates than others.

While all fly lines generally preferred to lay on yeast-inoculated media over sterile media (Fig 4), there was significant variation in oviposition response between fly lines (S3 Table). For example, the two fly lines derived from the fermentation tanks, FermA and FermB, exhibited conflicting oviposition responses despite being collected at the same time. CellarA and PPA, which were collected in a different winery, winery area, and time, exhibited more similar, intermediate oviposition behavior. These data show that while there is natural heterogeneity in yeast volatile sensitivity between fly lines, the variation in ovipositional preference is not specific to winery area.

#### Larval development

While oviposition substrate is important for larval fitness, larval development success and time is an indicator of nutritional health. *Drosophila* larva eclose both faster and more successfully when larval diet is supplemented with yeast [10,11]. To test if the yeasts associated with *Drosophila* in different areas of the winery have effects on larval development, we measured the development of winery fly lines when fed active monocultures of the yeast panel.

In corroboration with previous studies, we found that all fly lines develop more successfully on a diet supplemented with either live or dead yeast than on sterile media (S4 Fig and S4 Table). In fact, larvae grown on sterile media did not pupate at all, except for larvae from a single fly line, PPA, which only exhibited a 20% eclosion success on sterile media.

**Fig 5:**
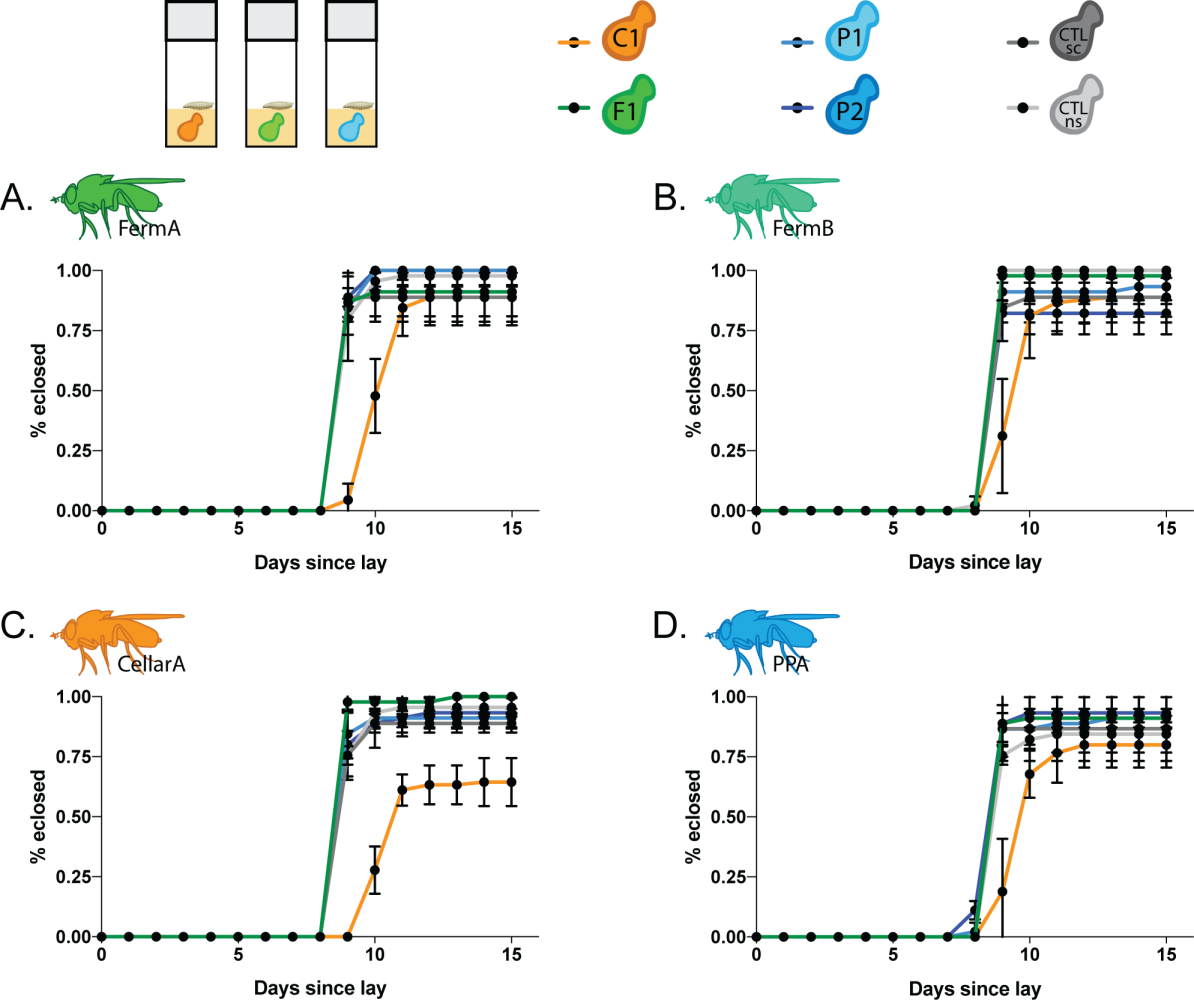
Some yeasts are more suitable for *Drosophila* development but do not follow a winery area specific pattern. Short, horizontal black lines represent standard deviation and black points represent the mean of all trials. Accompanying statistics for larval development time in S5 Table. Larval development of fly lines (A) FermA (B) FermB. (C) CellarA. (D) PPA.

If a fly-yeast specificity existed in the winery, we hypothesized that larvae would develop faster and more successfully when supplemented with yeast species that are overrepresented in the fungal communities vectored by *Drosophila* in their winery area. For the subsequent statistical analyses, we omitted the no yeast and dead yeast controls from our dataset, as we were only interested in differential effects of the yeast species in our panel. We found that all yeast species on the yeast panel were equally suitable for *Drosophila melanogaster* development, with the exception of CellarA on *Pichia kluyveri* (yeast isolate C1, Fig 5A). Only 64.4% of CellarA larvae supplemented with *Pichia kluyveri* successfully eclosed compared to greater than 88% successful eclosion on all other yeast species in the panel (S4 Table, p<0.0001 when compared to all other yeast species on panel). Interestingly, *Pichia kluyveri* did not have a significantly different effect on eclosion success in other fly lines tested.

Similar to the eclosion success, we found no significant differences in median development time between larva from all fly lines raised on active monocultures of the yeast species on our yeast panel except for *Pichia kluyveri* (Fig 5, Table 2). While the median developmental time of all four fly lines on other yeast species was nine days, larva raised on *Pichia kluyveri* had a delayed development time of 10–11 days (S5 Table).

**Table 2:** *Drosophila* larval development time from lay to eclosion when diet is supplemented with a single yeast species.

Fly Line	Yeast treatment	No. of larva	Median time to eclosion, days
FermA	C1	80	10
FermA	F1	41	9
FermA	P1	45	9
FermA	P2	45	9
FermA	CTLsc	40	9
FermA	CTLns	44	9
FermB	C1	80	10
FermB	F1	44	9
FermB	P1	42	9
FermB	P2	37	9
FermB	CTLsc	40	9
FermB	CTLns	45	9
CellarA	C1	58	11
CellarA	F1	45	9
CellarA	P1	41	9
CellarA	P2	42	9
CellarA	CTLsc	40	9
CellarA	CTLns	43	9
PPA	C1	72	10
PPA	F1	41	9
PPA	P1	41	9
PPA	P2	42	9
PPA	CTLsc	39	9
PPA	CTLns	38	9

Accompanying statistics in S5 Table.

In our the behavior assays we described above, we showed that *Pichia kluyveri* is both more attractive to adult flies and preferred as an oviposition substrate compared to other yeasts in the panel. So it is surprising that it has a negative effect on developmental timing. In addition, CellarA, a fly line established from a single female collected in a cellar, was the only line in which eclosion success was negatively affected by *Pichia kluyveri*, which is commonly vectored by *Drosophila* in cellars. Together, these data suggest effects of yeast on *Drosophila* larval development are nonspecific to winery area. Instead, a broad range of yeasts are suitable for *Drosophila* larval development and while most are equally beneficial, some are less favorable than others.

#### Longevity

**Fig 6:**
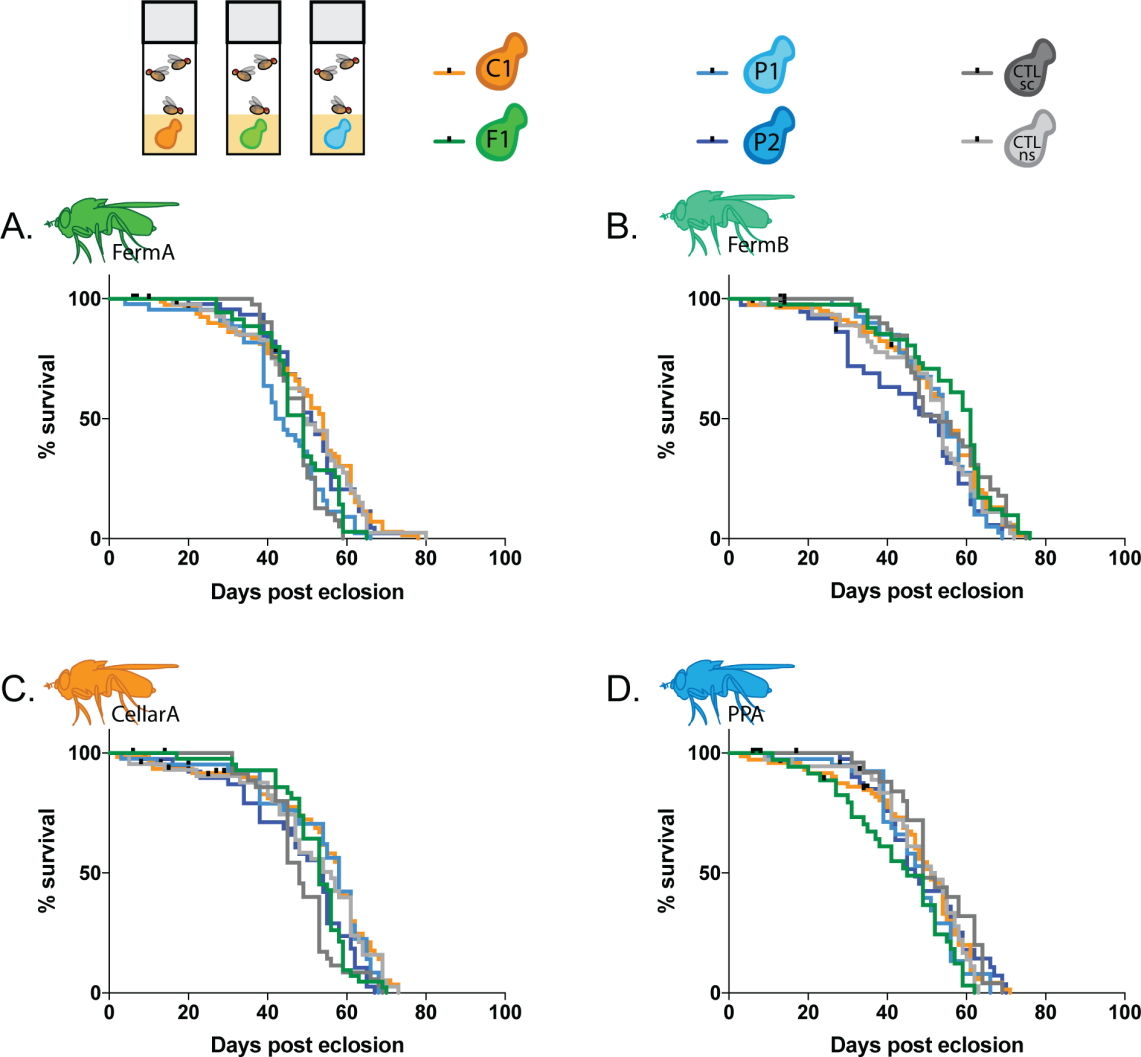
Fly-associated yeasts have no differing effects on *Drosophila* longevity. Accompanying statistics for longevity assay in S6 Table. Lifespan of fly lines (A) FermA (B) FermB. (C) CellarA. (D) PPA.

Finally, we tested if the yeast species in our panel had winery area specific effects on *Drosophila* lifespan. Supplementing diet with yeast can rescue undernutrition and extend lifespan in *D*. *melanogaster* [39]. To measure if winery area yeasts have differential effects on *Drosophila* longevity, we maintained the adult flies that eclosed from the previous larval development assay on sterile media inoculated with the same yeast species throughout the lifetime of the fly. While these wild fly lines exhibit natural variation in lifespans (ANOVA, p<0.0001, S6 Table), no particular yeast species had a significant effect on lifespan for a given fly line (Fig 6, Table 3). These results mirror those of the previously described olfactory preference, oviposition, and larval development assays.

**Table 3:** *Drosophila* lifespan when monoassociated with a single yeast species.

Fly Line	Yeast treatment	No. of flies	Median lifespan, days
FermA	C1	78	54
FermA	F1	35	49
FermA	P1	44	43
FermA	P2	44	51
FermA	CTLsc	40	49
FermA	CTLns	40	50
FermB	C1	75	55
FermB	F1	41	61
FermB	P1	40	55
FermB	P2	35	51
FermB	CTLsc	39	53
FermB	CTLns	45	54
CellarA	C1	57	58
CellarA	F1	42	53
CellarA	P1	36	58
CellarA	P2	38	54
CellarA	CTLsc	35	48
CellarA	CTLns	38	56
PPA	C1	70	51
PPA	F1	33	45
PPA	P1	38	49
PPA	P2	32	47
PPA	CTLsc	25	49
PPA	CTLns	36	51.5

Overall, we found that while different *Drosophila* lines vary in their behaviors towards some yeasts over others, these behaviors are not specific to winery area. These results consistently indicate that the structure in fungal communities vectored by *Drosophila* in different winery areas is not a direct result of fly behavior.

## Discussion

The interaction between fruit flies and yeast provides an ideal system in which to explore the parameters governing a natural mutualism. In the laboratory, *Drosophila melanogaster* exhibit a remarkable ability to discriminate between different strains of *Saccharomyces cerevisiae* based solely on volatile profile [17,19] but whether these preferences are relevant in nature is less clear.

Our first goal in conducting this study was to determine if the fungal communities associated with *Drosophila* varied in a predictable way among the different fly-rich habitats in wineries. Our data clearly demonstrate that they do, with different abundances of a generally shared set of yeast species serving as a signature of fermentation tanks, cellars, and pomace piles.

This observation raises the more interesting question of how distinct fly-associated fungal communities are determined. There are two possible, not mutually exclusive, explanations: flies could be selectively sampling fungi from these environments or flies could be passively sampling from environments with markedly different fungal populations.

Using a set of well-defined *Drosophila* behavioral assays, we found that the yeast species on our panel were generally attractive based on volatile cues, equally suitable for larval development, and have no differential effects on lifespan. Flies did not selectively sample the yeast species distinguishing the fungal communities of their winery habitats, suggesting that the stratification fly-associated yeast communities with habitat is not a direct result of fly behavior.

The alternative explanation is that flies passively sample fungal communities that are predetermined by the growth conditions and activity in a given winery habitat. Environmental factors, such as substrate nutrient composition and temperature, likely have a pronounced effect on yeast community composition. Wine production and the movement of processing equipment also affects the rate and time at which particular fungal species flow in and out of these environments. We cannot reject the hypothesis that passive sampling of the fungi that grow optimally in particular winery niches accounts for the observed structure in fly-associated fungal communities.

Even if flies do not deliberately associate with particular yeast species within wineries, they likely influence the overall yeast species composition in these environments. Many observations, including this study, have shown that flies carry diverse fungal populations, and that they can transfer these populations to new substrates [13,25,27]. It is possible that the yeast species tested here are commonly found in vineyards and wineries because of flies’ general attraction to them. Interestingly, we found that winery *Drosophila* were associated with many non-*Saccharomyces* wine yeasts that are known to contribute aroma complexity to wine fermentations [21,22,35], suggesting that flies may play a role in microbial terroir of wine flavor and aroma.

Our observations indicate that a broad range of yeast species are beneficial to *Drosophila* and can serve adequately as a mutualistic partner. However, lack of specificity does not imply that the fly-yeast mutualism is a weak interaction. The function and maintenance of a mutualism requires each partner to constantly evolve traits that allow for more efficient interactions with the other [40–42].

In a constantly fluctuating environment, where there are seasonal changes in food sources and temperature throughout the year, it is important that *Drosophila* be able to locate and subsist on many yeast species. Conversely, yeast must be able to produce volatile cues that ensure a close association with flies. Many of the yeast species tested in this study, despite being phylogenetically distant, share the ability to attract flies, confirming recent work demonstrating that volatile attractant production is a conserved trait across many yeast species [14]. However, the ability to attract flies does not always confer a benefit to fly fitness. In this study, we observed a yeast species, *Pichia kluyveri*, that was more attractive in the olfactory preference and oviposition assays than other yeasts but was the only yeast species that had a negative impact on larval fitness.

Wineries are habitats where both flies and yeast co-occur in large numbers and while this was advantageous for the goals of our study, we recognize that the yeast species tested in this study have already undergone selection for successful growth in a specific niche. The yeast used in this study were all commonly vectored by *Drosophila* in wineries. Whether flies are associated with the same yeast species in habitats where resources are scarce or habitats with other fruit substrates, such as apple orchards, remains open to investigation. Characterizing fly behavior towards yeast that are not associated with *Drosophila* or associated with *Drosophila* from very different habitats, would reveal whether the fly-yeast interactions we observed in this study are only relevant for this particular niche or more broadly applicable to the fly-yeast mutualism across many environments. Additionally, a comparison of the volatile profiles of these yeast species with those tested in this study could also elucidate whether the responses we observed are context-dependent and be used to identify specific compounds that influence fly behavior. We hope that future studies will continue to study the fly-yeast mutualism in other natural ecosystems to yield more insight into the parameters constraining this mutualism.

## Materials and methods

### Field collections of *Drosophila* and yeasts

All the wineries participating in this study practice organic farming and use spontaneous fermentations in winemaking. Adult *Drosophila* were collected in individual, sterile vials by direct aspiration or netting in cellars, fermentation tanks, and pomace piles. In 2015, flies were collected from two wineries in Healdsburg, California (HLD1) and the Santa Cruz Mountains, California (SMC) every two weeks from May 2015 –November 2015. During the 2016 harvest season, flies were collected from all four winery sites (Fig 1A) at a single time point from late September to early October. Geographical coordinates for collection sites were as follows: HLD1: 38°38'55.2"N 122°53'36.6"W, HLD2: 38°36'01.2"N 122°53'30.6"W, EBO: 37°53'27.7"N 122°10'55.0"W, and SCM: 37°18'18.2"N 122°07'44.2"W. Flies were immediately transported back to the laboratory and processed within four hours of collection. Upon arrival at the laboratory, we documented sex and grouped samples into either *D*. *melanogaster/D*. *simulans* or other *Drosophila* species by eye.

### Isolation and identification of yeasts for behavior assays

Roughly one third of the total flies collected were cooled in individual vials on ice for two minutes to reduce activity and placed onto 5% YPD agar plates (S7 Table). Roughly equal numbers of males and females were sampled. Flies were allowed to walk on plates overnight at ambient room temperature to deposit yeasts on plates and were aspirated off of plates in the morning. Yeast deposited on the plates were allowed to grow at ambient room temperature for 3–4 days. Single colonies, representing every yeast morphology present, were picked by eye and streaked onto fresh 5% YPD agar plates. If plates were overgrown with mold or single colonies were unable to be picked, a subsequent isolation was performed on a fresh plate. Isolated colonies were allowed to grow at ambient room temperature and stored at 4°C until molecular identification. Original plates were kept for an additional three days after picking to ensure slower growing yeast were sampled.

Yeast colonies were identified by Sanger sequencing of the internal transcribed spacer region (ITS) [31]. Colony PCR reactions were performed in 25uL reaction volumes as follows: 12.5uL GoTaq Colorless Master Mix (Promega), 2uL of ITS1 and ITS4 primer at 10uM, 8.5uL nuclease-free water (Promega), and colony spike-in. Reaction conditions were as follows: 95°C for 5 min followed by 35 cycles of 95°C for 30 s, 53°C for 30 s, and 72°C for 60 s, and a final extension of 72°C for 4 min (Jeremy Roop, personal communication). Amplification was verified on an agarose gel before being sent for Sanger sequencing (ELIM Biopharmaceuticals). Resulting sequences were trimmed for quality and then identified using BLAST (NCBI). Hits with an identity score greater than 98% were documented. After positive identification, yeast isolates were frozen as -80°C glycerol freezer stocks using standard protocol (Methods in Yeast Genetics, 2005) until use in behavior assays.

### Establishment of isofemale fly lines for behavior assays

Female *Drosophila* used for the yeast collection described above were aspirated onto fresh fly media (standard cornmeal-molasses-yeast medium, Bloomington Drosophila Stock Center) and used to establish isofemale lines. Each line underwent three rounds of intensive inbreeding where one to three virgin females were mated to three male siblings. After intensive inbreeding, lines were inbred at least 25 generations (10–40 female and male siblings) before being used in behavior assays.

### DNA extraction for amplicon study

The remaining flies collected (about 2/3 of flies) were immediately frozen and stored in individual, sterile 1.5mL microtubes for amplicon analysis. DNA was extracted following QIAGEN’s QIAamp Micro Kit tissue protocol with the modifications briefly described below [43]. After the overnight digestion with proteinase K, samples were bead beat (0.5 mm Zirconium beads, Ambion) in 200uL of WLB (S7 Table). The samples were beat beat (MoBio) twice for one minute at 4°C with a 30 second break in between, spun five minutes at ~14,000xg, and the supernatant was transferred to a new tube. Beads were resuspended in 1ml of buffer WLB and beat again an additional minute, spun down, and supernatant was pooled. Finally, beads were washed once more with 1mL of buffer WLB, spun down, and supernatant was pooled. The pooled supernatant was spun down to pellet any beads and transferred to a clean microtube. One ug of carrier RNA (QIAGEN) dissolved in buffer AE was added to the supernatant before proceeding to ethanol precipitation and elution per the manufacturer’s protocol. DNA samples were quantified (Qubit dsDNA HS assay kit, ThermoFisher Scientific) and stored at -20°C.

### Amplification and library construction

Fungal communities were characterized by amplifying the universal fungal internal transcribed spacer region I (ITSI) using BITS and B58S3 primers designed by Bokulich and Mills [32]. Each forward BITS primer includes a unique 8bp barcode connected to the universal forward primer with a 2bp linker sequence (sequences generously provided by Bokulich and Mills).

The following pre-PCR steps were carried out in a biosafety cabinet. The biosafety cabinet and laboratory supplies used were cleaned at the start of each day as follows to minimize PCR contamination: 10% bleach for 20mins, rinsed with autoclaved MilliQ water, 3% hydrogen peroxide for 10mins, and UV lamp for at least 5mins. PCR reactions were carried out in triplicate following the protocol previously used in [34] and described below.

For a single PCR reaction, reagents were added in the following order: 12.5uL GoTaq Colorless Master Mix (Promega), 2uL of B58S3 primer at 10uM, 5.5uL nuclease-free water (Promega), 2uL of BITS primer at 10uM, and finally 5–100 ng DNA template. Reaction conditions were as follows: 94°C for 3 min, 35 cycles of 94°C for 45s, 50°C for 60s, and 72°C for 90s, and finally extension of 72°C for 10 min [34]. PCR reactions were performed in 96 well plates with sample columns randomized between replicates to control for potential well biases. Both positive mock cultures (based on design described in [44], S8 Table) and negative controls (from extraction and amplification steps) were randomized in the PCR plates among real samples.

PCR replicates were pooled and cleaned using AMPure XP magnetic beads according to manufacturer’s protocol (Beckman Coulter). Pooled PCR products were quantified using the Qubit dsDNA HS assay kit and the samples on a single PCR plate were pooled at 30ng equimolar concentration. Any samples with concentrations of <1ng/uL were omitted. Negative control samples with concentrations of <1ng/uL were pooled to the largest volume of real samples. After pooling, each pool was cleaned and concentrated according to the manufacturer’s protocol (Zymo Clean and Concentrator), eluted in 22uL of sterile water, and quantified with the Qubit dsDNA HS assay kit.

Illumina sequencing libraries were prepared for each PCR plate pool using TruSeq RNA v2 kit (Illumina) beginning at the A-Tailing step of the manufacturer’s protocol using at least 200ng of starting material. A different Illumina index was used for each PCR plate pool. Libraries were verified and quantified using the Qubit dsDNA HS assay kit and the Agilent 2100 Bioanalyzer (Agilent Technologies). Up to four libraries were pooled and sequenced on a single 250bp paired-end Illumina MiSeq lane. Samples from 2015 were sequenced at the Vincent J. Coates Genomics Sequencing Laboratory (UC Berkeley) and the 2016 samples were sequenced at the UC Davis DNA Technologies Core. To control for the change in sequencing services, we resequenced a library from 2015 at UC Davis with 2016 samples and achieved very replicable results (S8 Table and S6 Fig).

### Data analysis of amplicon study

We followed many of the same processing steps outlined in [35,45] as we used the primers designed in these studies and expected the fungal communities of *Drosophila* in wineries to be similar to the fungal communities in wineries and breweries. Raw and quality filtered sequence counts from the following steps are summarized in S1 Table. Raw read pairs were merged using BBMerge (https://jgi.doe.gov/data-and-tools/bbtools/), demultiplexed in QIIME v1.9.1 [46], and primer sequences were trimmed using cutadapt [47]. Resulting reads were quality filtered in QIIME as follows: any read less than 80bp was removed, any read with more than 3 consecutive bases with a quality score <19 was removed, and any chimeric sequences were filtered against the UCHIME chimera reference dataset v7.1 [48] using the union method.

Open reference OTU picking was performed in QIIME using the UCLUST method [49] against a modified UNITE database [50,51] with a threshold of 97% pairwise identity. Sequence alignment and treebuilding were suppressed and taxonomy was assigned using BLAST (NCBI). After OTU picking, positive control mock culture samples and OTUs with ‘no blast hit’ were filtered. Using R version 3.2.4 [52], negative controls were removed, max sequence counts of all negative control OTUs were calculated, and then subtracted from all real samples to account for spurious sequences produced from possible PCR, sequencing, or spillover contamination [44,53]. Finally, an OTU threshold of 0.001% was applied [35,45].

Alpha diversity measurements of observed OTU richness were calculated and visualized in QIIME to reveal that all samples had been sequenced to saturation (S1 Fig). Community analyses were conducted using the vegan [54] and biom [55] packages in R. To determine relationship between the fungal communities vectored by *Drosophila*, samples were evenly subsampled to 1000 reads per sample and Bray-Curtis dissimilarity was calculated and visualized with nonmetric multidimensional scaling (NMDS) using ggplot2 [56]. ADONIS was used to calculate the relative effects of factors that distinguished fly-associated communities from others. The fungal communities associated with *Drosophila simulans*/*Drosophila melanogaster* samples were significantly different than those of other *Drosophila* species (S2 Fig) so other *Drosophila* samples were filtered out (S1 Data).

The relative abundances of fungal taxa in different winery areas were clustered using hierarchical clustering by taxa (Cluster 3.0) and visualized in Java TreeView and Prism 7 (GraphPad). To identify fungal taxa with significantly different relative abundances between winery areas, OTUs were collapsed by species name and the Kruskal-Wallis test was employed (with Bonferroni correction) in QIIME.

All raw reads are available from the NCBI Sequence Read Archive under accession SRP136413. Accompanying metadata information can be found in S3 Data.

### Olfactory preference assay

A custom trap-based olfactory preference assay previously designed by Schiabor et al was used to measure *Drosophila* olfactory preference [17]. Yeast species were plated onto agar grape juice plates (S7 Table) and grown at 30°C for 22 hours. The following day, plates were removed from the incubator, fitted with a custom printed lid, and secured with Parafilm. Lids were topped with a 50mL conical centrifuge tube (Falcon) with the end removed and covered in mesh. A funnel was fashioned from 150mm filter paper (Whatman, 150mm, Grade 1) with a 5mm hole snipped off the tip and secured to the top of the centrifuge tube with tape.

*Drosophila melanogaster* lines were raised at room temperature (21°C-23°C) on standard cornmeal-molasses-yeast media and aged at room temperature for at least four days under ambient lighting conditions (i.e. adjacent to a window) before being used in behavior assays. One hundred and twenty 4–10 day old mixtures of male and female flies were anesthetized with CO_2_ and allowed to recover on cornmeal-molasses-yeast media for two hours before being used in behavior assays.

Pairwise comparisons of yeast were used to assay for olfactory preference. Two traps for each yeast species were placed into behavior arenas (*Drosophila* population cages, 24” x 12” clear acrylic cylinders, TAP plastics) and fitted with netting (Genesse Scientific) as shown in Figs 3 and S5A. All four possible orientations of plates within the arena were tested to control for potentially confounding environmental variables such a light (S5B Fig).

Flies were introduced into behavior arenas at 3pm and allowed survey traps. After 18 hours, traps were removed from the arena and the number of flies in each trap were counted, sexed, and recorded. Flies were only used in behavior assays once and were discarded after counting. A preference index was calculated from the number of flies in each trap as follows:

ForA=totalnumberoffliesintrapsbaitedwithYeastA

ForB=totalnumberoffliesintrapsbaitedwithYeastB

$$Preference\;Index=\frac{\left(A-B\right)}{\left(A+B\right)}$$

A positive PI indicates a preference for yeast A, a negative PI indicates a preference for B, and a PI of 0 indicates no preference. Multiple t-tests with Bonferroni correction were executed in Prism 7 and used determine which yeast preferences were significant.

### Oviposition assay

The egg laying assay in this study was adapted from Joseph et al 2009 [57] and Fischer et al 2017 [58]. At 10am, 75mL of sterile liquid grape juice (S7 Table) was inoculated with 1.5mL of yeast cells diluted to OD_600_ = 1 in sterile 1X PBS (Mediatech Inc). These cultures, and a negative control, were grown shaking at 30°C for 72 hours.

Oviposition assay cages were fashioned from polypropylene *Drosophila* bottles (6oz, square, Genesse Scientific) with the bottom cut out and covered with mesh. During acclimation, cages were capped petri dishes (35x10mm, Falcon) filled with grape agar premix (Genesse Scientific) and topped with yeast paste (Red Star).

Similar to olfactory preference assays, *Drosophila melanogaster* lines were raised and aged for four to ten days at room temperature on standard cornmeal-molasses-yeast media. Twenty-four hours before the assay began, twenty non-virgin females were acclimated to oviposition cages at 25°C. Cages were kept on the top shelf of a 25°C incubator on a 12 hour light cycle, placed on the side, and positioned so the mesh bottom faced the back of the incubator and the plate faced the door of the incubator (S5C Fig).

At least one hour before the start of the behavior assay, acclimated cages were cleared by replacing the petri dish with a new grape agar premix plate without yeast paste and returned to 25°C. After 72 hours of fermentation, cultures were removed from 30°C, mixed 1:1 with a boiled water-agar solution (BD Bacto Dehydrated Agar) cooled to 65°C, to achieve a final agarose concentration of 1.6%. The lids of petri dishes (35x10mm, Falcon) were divided in half using laminated paper. Plates containing half uninoculated grape juice and half inoculated grape juice were created by pouring both sides simultaneously (S5C Fig).

Plates were cooled for 15 minutes at room temperature and the laminated paper was removed. Plates were immediately used in behavior assays by replacing the grape agar premix plate cage topper. *Drosophila* females were allowed to oviposit on plates for three hours before plates were removed for counting (usually from around 12noon to 3pm). Flies were only used once and discarded at the end of the assay.

An oviposition index (OI) was calculated from the number of embryos deposited on each side of the plate as follows:

ForY=totalnumberofeggsovipositedoninoculatedside

$$Preference\;Index=\frac{\left(A-B\right)}{\left(A+B\right)}$$

$$Oviposition\;Index=\frac{\left(Y-N\right)}{\left(Y+N\right)}$$

A positive OI indicates an oviposition preference for the yeast side, a negative OI indicates a preference for the control side, and a OI of 0 indicates no preference for either side. Multiple t-tests with Bonferroni correction were used to determine whether the yeast tested elicited a significantly different oviposition preference in relation to the control. One way ANOVA was used to test for any differences in ovipostion responses between fly lines for a given yeast species. If ANOVA results were statistically significant, Tukey's multiple comparisons test was used to identify the fly lines exhibiting ovipostion responses that were significantly different than other lines. These statistical analyses and those described in the methods following were implemented in Prism 7 with a significance cutoff of p>0.05.

### Gas chromatography–mass spectrometry (GC-MS)

A subset of the oviposition plates used in the oviposition assays were also sampled by GC-MS in parallel with the behavior assays using a stirbar sorptive extraction (SBSE) and thermal desorption approach. Oviposition plates were placed in sterile 60 x 15mm petri dishes for headspace sampling. As previously described in [17], a conditioned, Twister stir bar (10 mm in length, 0.5mm film thickness, 24uL polydimethylsiloxane, Gerstel Inc) was suspended from the lid of the larger petri dish with rare earth magnets for 40 minutes at room temperature. The Twister bar was then dried using a Kimwipe, placed in a thermal desorption sample tube, topped with a transport adapter, and loaded onto sampling tray (Gerstel Inc).

Automated sampling and analysis was performed using the Gerstel MPS system and MAESTRO integrated into Chemstation software. Sample analysis was performed on an Agilent Technologies 7890A/5975C GC-MS equipped with a HP-5MS (30m × 0.25mm, i.d., 0.25micrometers film thickness, Agilent Technologies) column.

Samples were thermally desorbed using the Gerstel Thermal Desorption Unit (TDU) in splitless mode, ramping from 30°C to 250°C at a rate of 120°C/min, and held at the final temperature for 5 minutes. The Gerstel Cooled Injection System (CIS-4) was cooled to -100°C with liquid nitrogen before ramping to 250°C at a rate of 12°C/min and held for 3 mins for injection into the column. The injector inlet was operated in the Solvent Vent mode, with a vent pressure of 9.1473 psi, a vent flow of 30mL/min, and a purge flow of 6mL/min.

The GC oven temperature program was set to 40°C for 2 min, raised to 140°C at 4°C/min, and finally raised to 195°C at 15°C/min and held for 10 min. A constant helium flowrate of 1.2 mL/min was used as carrier gas. The MSD transfer line temperature was set at 280°C. The MS was operated in EI mode with the electron voltage set at autotune values. The detector was set to scan from 30 to 300amu at a threshold of 150 at a scanning rate of 2.69 scans/second. The ion source and quadrupole temperatures were set at 230°C and 150°C, respectively.

GC-MS data files were visually inspected using Chemstation and peaks were identified using the NIST O8 database. Datafiles were transferred, parsed, and analyzed using custom written Matlab scripts in [17]. Every chromatogram trace represents, at minimum, the average of 6 replicates.

### Larval development assay

In order to test the effects of each yeast species on larval development time and success, *Drosophila* larvae were raised on sterile, yeast-free media supplemented with a single yeast species. As in the previously described assays, *Drosophila melanogaster* lines were raised and aged for four to ten days at room temperature on standard cornmeal-molasses-yeast media. Twenty-four hours before embryo collection, at least 50 adults flies were acclimated to the oviposition assay cages capped with grape agar premix plates and yeast at 25°C as described above.

At 9am the following morning, plates in oviposition cages were replaced with new grape agar premix with yeast plates to clear any hoarded eggs. After 30 minutes, clearing plates were replaced with new grape agar premix with yeast plates and flies were allowed to lay for two hours at 25°C for embryo collection. After two hours, collection plate was removed and aged at 25°C for two hours. Aged embryos were washed off plates with MilliQ water into a embryo wash basket fashioned out of a 50mL conical (Falcon) with the end cut off and the top of the lid removed and covered with thin mesh. In the wash basket, embryos were dechorionated with 30% bleach for three minutes, consequently removing any previously associated yeast. Embryos were washed with sterile, autoclaved MilliQ and then with sterile PBS-t (1X PBS, 0.5% triton). Using a sterile paintbrush, embryos were moved onto sterile agar plates and allowed to hatch at 25°C overnight. For data analyses, this day was considered Day 0 of the assay.

On the same day at 9:30am, 5mL starter cultures of liquid 5% YPD (S7 Table) were inoculated with the yeast species of interest and grown shaking at 30°C. At 3:30pm, cultures were removed and diluted to OD_600_ = 0.5. *Drosophila* vials with sterile, yeast-free GB media (S7 Table) were spotted with 50uL of diluted culture and grown at 30°C overnight. Three replicate vials for each yeast species and each fly line were set up (S5E Fig).

At 11am the next day, embryo plates were removed from the incubator. Using a sterile paintbrush (dipped in 50% bleach, 75% ethanol, autoclaved MilliQ, and sterile PBS-t between each vial), 15 larvae were moved into each vial and allowed to develop at 25°C. Due to the extensive setup and time constrains of fly development, we tested the larval development of all four fly lines for each yeast species on the panel in three groups over 2 months. In each group, a positive control on standard cornmeal-molasses-yeast media (dead yeast) and a negative control on sterile GB media with no yeast supplement was run in parallel with experimental conditions.

We opted to start our assays with larvae instead of embryos in an effort to control for any death after dechorionation. For data analyses, this day was considered Day 1 of the assay. Each day, vials were checked for emerged adults and randomized within the incubator until the assay was terminated on Day 16. Adults that eclosed successfully were moved into sterile GB media vials daily and subsequently used for the longevity assays described below.

Eclosion curves and statistics were plotted in Prism 7. To test whether larvae given any yeast eclosed more successfully than those given no yeast and whether some yeast species resulted in greater eclosion success than others, one way ANOVA followed by Tukey's multiple comparisons test was used to calculate significance values for each yeast species and controls within each fly line.

### Longevity assay

To study the effects of each yeast species on the lifespan of *Drosophila*, adults that eclosed successfully from the larval development assay were fed a diet supplemented with the same yeast species throughout their lifetime. At 9am on Day 8 of the larval development assay, 5mL starter cultures of liquid 5% YPD were inoculated with the yeast species of interest and grown shaking at 30C. At 3:30pm, cultures were removed and diluted to OD_600_ = 0.5. *Drosophila* vials with sterile, yeast-free GB media (S7 Table) were spotted with 50uL of diluted culture and grown at 30°C overnight (S5E Fig).

As flies hatched off of the larval development assay, they were moved onto the inoculated media and checked every day. Each day, vials were randomized within the incubator to control for positional effects. Flies were maintained at 25°C and pushed onto fresh media twice a week, once into sterile GB media and once into inoculated media prepared as described above.

Survival curves and statistics were plotted in Prism 7. One way ANOVA followed by Tukey's multiple comparisons test was used to test whether different fly lines had significantly different lifespans. The effect of single yeast species on the lifespan of a single fly line was tested using one way ANOVA but found no significant differences.

## Supporting information

S1 Fig Alpha diversity richness QIIME rarefaction curves. (PNG)

S2 Fig The fungal communities vectored by *Drosophila melanogaster/Drosophila simulans* are different than other *Drosophila* species. Bray-Curtis dissimilarity NMDS of fungal communities vectored by *Drosophila* in all vineyards in 2015 and 2016. Each sample was rarefied to 1000 sequences and is represented by a single point, color-coded by species. ADONIS: R^2^ = 0.018, *p* = 0.001.
(TIF)

S3 Fig *Pichia manshurica* (yeast isolate P2) does not ferment well in liquid grape juice. When measured by GC-MS, *Pichia manshurica* produces very low levels of ethanol (inset) and other volatile metabolites compared to other yeast species on the panel. Each line represents the average of eight GC-MS replicates for a given yeast species. Replicates were sampled for GC-MS in parallel with oviposition assays.
(TIF)

S4 Fig *Drosophila* larvae supplemented with any species yeast species, dead or alive, develop more successfully than those fed no yeast at all. Note that data for larvae that were not given yeast only exist for PPA line because no larvae eclosed without the addition of yeast in any other lines.
(TIF)

S5 Fig Photographs of behavior assays. (A) Setup of a single, trap-based olfactory assay. (B) Close up of trap-based olfactory assay. Traps can be arranged in four possible combinations, two of which are depicted here. (C) Setup of six oviposition assays. (D) Example agar plate after oviposition assay. Left side is uninnoculated grape juice agar, right side is yeast inoculated grape juice agar. (E) Larval development and longevity assays were performed in wide vials shown here. Both larvae and adults were exposed to a live monoculture of yeast spotted onto sterile banana media. Depicted are Day 7, negative control vials that had no larvae or adult flies but grew alongside behavioral assays to monitor bacterial or mold contamination.
(TIF)

S6 Fig Bray-Curtis dissimilarity NMDS of PCR plate sequenced with samples collected in 2015 at UC Berkeley and the same plate resequenced with samples collected in 2016 at UC Davis. Each sample was rarefied to 200 sequences and is represented by a single point, color-coded by year.
(TIF)

S1 Table Library and quality filtering statistics. (PDF)

S2 Table Alpha richness of fungi vectored by *Drosophila* by vineyard and harvest season. Rarefied to 1000 sequences per sample.
(PDF)

S3 Table Statistics accompanying the ovipostion index responses of each fly line in Fig 4 for each yeast species. Each table represents a different yeast species. Within each yeast species, ANOVA was first used to test for any differences in ovipostion responses between fly lines for a given yeast species and denoted by a * next to each yeast isolate. If ANOVA results were statistically significant, Tukey's multiple comparisons test was used to identify the fly lines exhibiting ovipostion responses that were significantly different than other lines and are depicted with * within the table. ns: not significant, *: p<0.05, **: p<0.01, ***: p<0.001, ****: p<0.0001.
(PDF)

S4 Table Average percentage of *Drosophila* larvae that eclose successfully when developing on different yeast species. Control conditions are shaded grey. (ANOVA when compared to no yeast control followed by Tukey's multiple comparisons test was used to calculate significance values, ****: p < 0.0001).
(PDF)

S5 Table Statistics to accompany Table 2 on effects of yeast species on larval development time for each fly line. (One way ANOVA followed by Tukey's multiple comparisons test was used to calculate significance values, ****: p<0.0001).
(PDF)

S6 Table Comparisons of fly line lifespans to each other. One way ANOVA followed by Tukey's multiple comparisons test was used to calculate significance values, ns: not significant, *: p<0.05, **: p<0.01, ***: p<0.001, ****: p<0.0001.
(PDF)

S7 Table Media and buffers used in this study. (PDF)

S8 Table Components of mock community and sequencing results. Mock culture replicates were independent with the exception of "2016 reseq of 2015" which came from amplicons made in 2015 and resequenced in 2016 as a control. Yeast isolates used in mock cultures were isolated, species were identified, and DNA was extracted as described in Materials and Methods. The mock culture was created by combining 4uL of each yeast species listed below. The resulting mix was used as DNA template in the amplification protocol described.
(PDF)

S1 Data Processed OTU table. Following the filtering of fly samples that were not *D*. *melanogaster* or *D*. *simulans*.
(BIOM)

S2 Data Raw behavior assay data. For olfactory preference assay, oviposition assay, larval development assay, and longevity assay.
(XLSX)

S3 Data Sample key and metadata to accompany amplicon data. (XLSX)

## References

[1] McFall-Ngai M, Hadfield MG, Bosch TCG, Carey HV, Domazet-Lošo T, Douglas AE, et al Animals in a bacterial world, a new imperative for the life sciences. Proc Natl Acad Sci U S A. 2013;110: 3229–3236. doi: 10.1073/pnas.1218525110 23391737 10.1073/pnas.1218525110 PMC3587249

[2] Bäckhed F, Ding H, Wang T, Hooper LV, Koh GY, Nagy A, et al The gut microbiota as an environmental factor that regulates fat storage. Proc Natl Acad Sci U S A. 2004;101: 15718–15723. doi: 10.1073/pnas.0407076101 15505215 10.1073/pnas.0407076101 PMC524219

[3] Round JL, O’Connell RM, Mazmanian SK. Coordination of tolerogenic immune responses by the commensal microbiota. J Autoimmun. 2010;34: J220–5. doi: 10.1016/j.jaut.2009.11.007 19963349 10.1016/j.jaut.2009.11.007 PMC3155383

[4] Koch H, Schmid-Hempel P. Socially transmitted gut microbiota protect bumble bees against an intestinal parasite. Proc Natl Acad Sci U S A. 2011;108: 19288–19292. doi: 10.1073/pnas.1110474108 22084077 10.1073/pnas.1110474108 PMC3228419

[5] Nishino R, Mikami K, Takahashi H, Tomonaga S, Furuse M, Hiramoto T, et al Commensal microbiota modulate murine behaviors in a strictly contamination-free environment confirmed by culture-based methods. Neurogastroenterol Motil. 2013;25: 521–e371. doi: 10.1111/nmo.12110 23480302 10.1111/nmo.12110

[6] Lee PN, McFall-Ngai MJ, Callaerts P, de Couet HG. The Hawaiian bobtail squid (Euprymna scolopes): a model to study the molecular basis of eukaryote-prokaryote mutualism and the development and evolution of morphological novelties in cephalopods. Cold Spring Harb Protoc. 2009;2009: db.emo135. 10.1101/pdb.emo135 20150047

[7] Wilson ACC, Ashton PD, Calevro F, Charles H, Colella S, Febvay G, et al Genomic insight into the amino acid relations of the pea aphid, Acyrthosiphon pisum, with its symbiotic bacterium Buchnera aphidicola. Insect Mol Biol. 2010;19 Suppl 2: 249–258. 20482655 10.1111/j.1365-2583.2009.00942.x

[8] Hansen AK, Moran NA. Aphid genome expression reveals host-symbiont cooperation in the production of amino acids. Proc Natl Acad Sci U S A. 2011;108: 2849–2854. doi: 10.1073/pnas.1013465108 21282658 10.1073/pnas.1013465108 PMC3041126

[9] Broderick NA, Lemaitre B. Gut-associated microbes of Drosophila melanogaster. Gut Microbes. 2012;3: 307–321. doi: 10.4161/gmic.19896 22572876 10.4161/gmic.19896 PMC3463489

[10] Sang JH, King RC. Nutritional Requirements of Axenically Cultured Drosophila Melanogaster Adults. J Exp Biol. The Company of Biologists Ltd; 1961;38: 793–809.

[11] Anagnostou C, Dorsch M, Rohlfs M. Influence of dietary yeasts on Drosophila melanogaster life-history traits. Entomol Exp Appl. 2010;136: 1–11.

[12] Gilbert DG. Dispersal of yeasts and bacteria by Drosophila in a temperate forest. Oecologia. 1980;46: 135–137. doi: 10.1007/BF00346979 28310639 10.1007/BF00346979

[13] Reuter M, Bell G, Greig D. Increased outbreeding in yeast in response to dispersal by an insect vector. Curr Biol. 2007;17: R81–3. doi: 10.1016/j.cub.2006.11.059 17276903 10.1016/j.cub.2006.11.059

[14] Becher PG, Hagman A, Verschut V, Chakraborty A, Rozpędowska E, Lebreton S, et al Chemical signaling and insect attraction is a conserved trait in yeasts. Ecol Evol. doi: 10.1002/ece3.3905 29531709 10.1002/ece3.3905 PMC5838033

[15] Becher PG, Flick G, Rozpędowska E, Schmidt A, Hagman A, Lebreton S, et al Yeast, not fruit volatiles mediate Drosophila melanogaster attraction, oviposition and development. Thompson K, editor. Funct Ecol. 2012;26: 822–828.

[16] Becher PG, Bengtsson M, Hansson BS, Witzgall P. Flying the Fly: Long-range Flight Behavior of Drosophila melanogaster to Attractive Odors. J Chem Ecol. 2010;36: 599–607. doi: 10.1007/s10886-010-9794-2 20437263 10.1007/s10886-010-9794-2

[17] Schiabor KM, Quan AS, Eisen MB. Saccharomyces cerevisiae mitochondria are required for optimal attractiveness to Drosophila melanogaster. PLoS One. 2014;9: e113899 doi: 10.1371/journal.pone.0113899 25462617 10.1371/journal.pone.0113899 PMC4252075

[18] Palanca L, Gaskett AC, Günther CS, Newcomb RD, Goddard MR. Quantifying variation in the ability of yeasts to attract Drosophila melanogaster. Leulier F, editor. PLoS One. 2013;8: e75332 doi: 10.1371/journal.pone.0075332 24086510 10.1371/journal.pone.0075332 PMC3783394

[19] Christiaens JF, Franco LM, Cools TL, De Meester L, Michiels J, Wenseleers T, et al The Fungal Aroma Gene ATF1 Promotes Dispersal of Yeast Cells through Insect Vectors. CellReports. The Authors; 2014; 1–25. 10.1016/j.celrep.2014.09.009 25310977

[20] Lam SSTH Howell KS. Drosophila-associated yeast species in vineyard ecosystems. FEMS Microbiol Lett. 2015;362 doi: 10.1093/femsle/fnv170 26391524 10.1093/femsle/fnv170

[21] Ciani M, Comitini F, Mannazzu I, Domizio P. Controlled mixed culture fermentation: a new perspective on the use of non-Saccharomyces yeasts in winemaking. FEMS Yeast Res. 2010;10: 123–133. doi: 10.1111/j.1567-1364.2009.00579.x 19807789 10.1111/j.1567-1364.2009.00579.x

[22] Bokulich NA, Collins TS, Masarweh C, Allen G, Heymann H, Ebeler SE, et al Associations among Wine Grape Microbiome, Metabolome, and Fermentation Behavior Suggest Microbial Contribution to Regional Wine Characteristics. MBio. 2016;7 doi: 10.1128/mBio.00631-16 27302757 10.1128/mBio.00631-16 PMC4959672

[23] Mortimer R, Polsinelli M. On the origins of wine yeast. Res Microbiol. 1999;150: 199–204. 10229949 10.1016/s0923-2508(99)80036-9

[24] Stamps JA, Yang LH, Morales VM, Boundy-Mills KL. Drosophila Regulate Yeast Density and Increase Yeast Community Similarity in a Natural Substrate. Kytöviita M-M, editor. PLoS One. 2012;7: e42238 doi: 10.1371/journal.pone.0042238 22860093 10.1371/journal.pone.0042238 PMC3409142

[25] Goddard MR, Anfang N, Tang R, Gardner RC, Jun C. A distinct population of Saccharomyces cerevisiae in New Zealand: evidence for local dispersal by insects and human-aided global dispersal in oak barrels. Environ Microbiol. 2010;12: 63–73. doi: 10.1111/j.1462-2920.2009.02035.x 19691498 10.1111/j.1462-2920.2009.02035.x

[26] Stefanini I, Dapporto L, Legras J-L, Calabretta A, Di Paola M, De Filippo C, et al Role of social wasps in Saccharomyces cerevisiae ecology and evolution. Proc Natl Acad Sci U S A. 2012;109: 13398–13403. doi: 10.1073/pnas.1208362109 22847440 10.1073/pnas.1208362109 PMC3421210

[27] Chandler JA, Eisen JA, Kopp A. Yeast Communities of Diverse Drosophila Species: Comparison of Two Symbiont Groups in the Same Hosts. Appl Environ Microbiol. 2012;78: 7327–7336. doi: 10.1128/AEM.01741-12 22885750 10.1128/AEM.01741-12 PMC3457106

[28] Starmer WT, Fogleman JC. Coadaptation of Drosophila and Yeasts in Their Natural Habitat. J Chem Ecol. 1986;12: 1037–1055. doi: 10.1007/BF01638995 24307046 10.1007/BF01638995

[29] Dobzhansky T, Cooper DM, Phaff HJ, Knapp EP, Carson HL. Differential attraction of species of Drosophila to different species of yeasts. Ecology. JSTOR; 1956; 544–550.

[30] Barata A, Santos SC, Malfeito-Ferreira M, Loureiro V. New insights into the ecological interaction between grape berry microorganisms and Drosophila flies during the development of sour rot. Microb Ecol. 2012;64: 416–430. doi: 10.1007/s00248-012-0041-y 22438040 10.1007/s00248-012-0041-y

[31] Schoch CL, Seifert KA, Huhndorf S, Robert V, Spouge JL, Levesque CA, et al Nuclear ribosomal internal transcribed spacer (ITS) region as a universal DNA barcode marker for Fungi. Proc Natl Acad Sci U S A. 2012;109: 6241–6246. doi: 10.1073/pnas.1117018109 22454494 10.1073/pnas.1117018109 PMC3341068

[32] Bokulich NA, Mills DA. Improved selection of internal transcribed spacer-specific primers enables quantitative, ultra-high-throughput profiling of fungal communities. Appl Environ Microbiol. 2013;79: 2519–2526. doi: 10.1128/AEM.03870-12 23377949 10.1128/AEM.03870-12 PMC3623200

[33] Hoang D, Kopp A, Chandler JA. Interactions between Drosophila and its natural yeast symbionts—Is Saccharomyces cerevisiae a good model for studying the fly-yeast relationship? PeerJ. PeerJ Inc.; 2015;3: e1116 doi: 10.7717/peerj.1116 26336636 10.7717/peerj.1116 PMC4556146

[34] Bokulich NA, Ohta M, Richardson PM, Mills DA. Monitoring Seasonal Changes in Winery-Resident Microbiota. PLoS One. 2013;8: e66437 doi: 10.1371/journal.pone.0066437 23840468 10.1371/journal.pone.0066437 PMC3686677

[35] Bokulich NA, Thorngate JH, Richardson PM, Mills DA. Microbial biogeography of wine grapes is conditioned by cultivar, vintage, and climate. Proc Natl Acad Sci U S A. National Acad Sciences; 2014;111: E139–E148. doi: 10.1073/pnas.1317377110 24277822 10.1073/pnas.1317377110 PMC3890796

[36] Budick SA, Dickinson MH. Free-flight responses of Drosophila melanogaster to attractive odors. J Exp Biol. 2006;209: 3001–3017. doi: 10.1242/jeb.02305 16857884 10.1242/jeb.02305

[37] Richmond RC, Gerking JL. Oviposition site preference in Drosophila. Behav Genet. 1979;9: 233–241. 115458 10.1007/BF01071304

[38] Azanchi R, Kaun KR, Heberlein U. Competing dopamine neurons drive oviposition choice for ethanol in Drosophila. Proc Natl Acad Sci U S A. 2013;110: 21153–21158. doi: 10.1073/pnas.1320208110 24324162 10.1073/pnas.1320208110 PMC3876210

[39] Yamada R, Deshpande SA, Bruce KD, Mak EM, Ja WW. Microbes Promote Amino Acid Harvest to Rescue Undernutrition in Drosophila. Cell Rep. 2015; doi: 10.1016/j.celrep.2015.01.018 25683709 10.1016/j.celrep.2015.01.018 PMC4534362

[40] Law R, Koptur S. On the evolution of non-specific mutualism. Biol J Linn Soc Lond. Oxford University Press; 1986;27: 251–267.

[41] Ollerton J. Biological barter”: patterns of specialization compared across different mutualisms Plant-pollinator interactions: from specialization to generalization University of Chicago Press, Chicago 2006; 411–435.

[42] Thompson JN. The coevolving web of life (American society of naturalists presidential address). Am Nat. The University of Chicago Press; 2009;173: 125–140. doi: 10.1086/595752 19119876 10.1086/595752

[43] Elya C, Zhang V, Ludington WB, Eisen MB. Stable Host Gene Expression in the Gut of Adult Drosophila melanogaster with Different Bacterial Mono-Associations. PLoS One. 2016;11: e0167357 doi: 10.1371/journal.pone.0167357 27898741 10.1371/journal.pone.0167357 PMC5127555

[44] Glassman SI, Levine CR, DiRocco AM, Battles JJ, Bruns TD. Ectomycorrhizal fungal spore bank recovery after a severe forest fire: some like it hot. ISME J. 2015; doi: 10.1038/ismej.2015.182 26473720 10.1038/ismej.2015.182 PMC5029211

[45] Bokulich NA, Subramanian S, Faith JJ, Gevers D, Gordon JI, Knight R, et al Quality-filtering vastly improves diversity estimates from Illumina amplicon sequencing. Nat Methods. 2013;10: 57–59. doi: 10.1038/nmeth.2276 23202435 10.1038/nmeth.2276 PMC3531572

[46] Caporaso JG, Kuczynski J, Stombaugh J, Bittinger K, Bushman FD, Costello EK, et al QIIME allows analysis of high-throughput community sequencing data. Nat Methods. 2010;7: 335–336. doi: 10.1038/nmeth.f.303 20383131 10.1038/nmeth.f.303 PMC3156573

[47] Martin M. Cutadapt removes adapter sequences from high-throughput sequencing reads. EMBnet journal. journal.embnet.org; 2011; Available: http://journal.embnet.org/index.php/embnetjournal/article/view/200

[48] Nilsson RH, Tedersoo L, Ryberg M, Kristiansson E, Hartmann M, Unterseher M, et al A Comprehensive, Automatically Updated Fungal ITS Sequence Dataset for Reference-Based Chimera Control in Environmental Sequencing Efforts. Microbes Environ. 2015;30: 145–150. doi: 10.1264/jsme2.ME14121 25786896 10.1264/jsme2.ME14121 PMC4462924

[49] Edgar RC. Search and clustering orders of magnitude faster than BLAST. Bioinformatics. 2010;26: 2460–2461. doi: 10.1093/bioinformatics/btq461 20709691 10.1093/bioinformatics/btq461

[50] Abarenkov K, Henrik Nilsson R, Larsson K-H, Alexander IJ, Eberhardt U, Erland S, et al The UNITE database for molecular identification of fungi—recent updates and future perspectives. New Phytol. Wiley Online Library; 2010;186: 281–285. doi: 10.1111/j.1469-8137.2009.03160.x 20409185 10.1111/j.1469-8137.2009.03160.x

[51] Kõljalg U, Larsson K-H, Abarenkov K, Nilsson RH, Alexander IJ, Eberhardt U, et al UNITE: a database providing web-based methods for the molecular identification of ectomycorrhizal fungi. New Phytol. Wiley Online Library; 2005;166: 1063–1068. doi: 10.1111/j.1469-8137.2005.01376.x 15869663 10.1111/j.1469-8137.2005.01376.x

[52] RDevelopment CORE TEAM R, Others. R: A language and environment for statistical computing. R foundation for statistical computing Vienna, Austria; 2008.

[53] Nguyen NH, Smith D, Peay K, Kennedy P. Parsing ecological signal from noise in next generation amplicon sequencing. New Phytol. 2015;205: 1389–1393. doi: 10.1111/nph.12923 24985885 10.1111/nph.12923

[54] Oksanen J, Blanchet FG, Kindt R, Legendre P, Minchin PR, O’Hara RB, et al Vegan: community ecology package version 2.0–2. 2011. R CRAN package 2012;

[55] McDonald D, Clemente JC, Kuczynski J, Rideout JR, Stombaugh J, Wendel D, et al The Biological Observation Matrix (BIOM) format or: how I learned to stop worrying and love the ome-ome. Gigascience. 2012;1: 7 doi: 10.1186/2047-217X-1-7 23587224 10.1186/2047-217X-1-7 PMC3626512

[56] Wickham H. ggplot2: Elegant Graphics for Data Analysis. Springer; 2016.

[57] Joseph RM, Devineni AV, King IFG, Heberlein U. Oviposition preference for and positional avoidance of acetic acid provide a model for competing behavioral drives in Drosophila. Proc Natl Acad Sci U S A. 2009;106: 11352–11357. doi: 10.1073/pnas.0901419106 19541615 10.1073/pnas.0901419106 PMC2698888

[58] Fischer CN, Trautman EP, Crawford JM, Stabb EV, Handelsman J, Broderick NA. Metabolite exchange between microbiome members produces compounds that influence Drosophila behavior. Elife. 2017;6 doi: 10.7554/eLife.18855 28068220 10.7554/eLife.18855 PMC5222558

