# Supplementary figures and images for: The ecology of the Drosophila-yeast mutualism in wineries
Source: PLoS One. 2018 May 16;13(5):e0196440. doi: 10.1371/journal.pone.0196440 (PMC5955509; doi:10.1371/journal.pone.0196440)

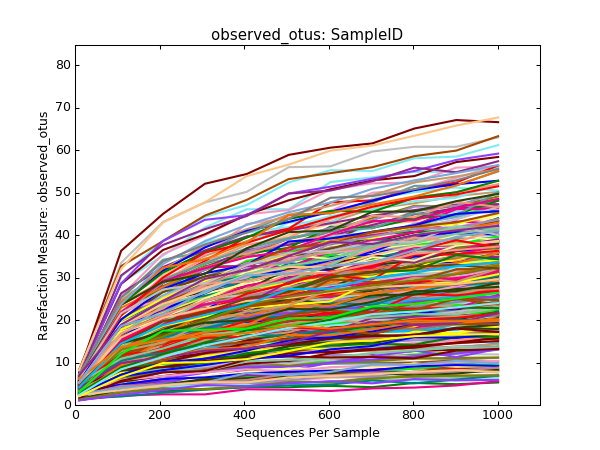

Supplement: S1 Fig — (PNG) [file pone.0196440.s001.png]

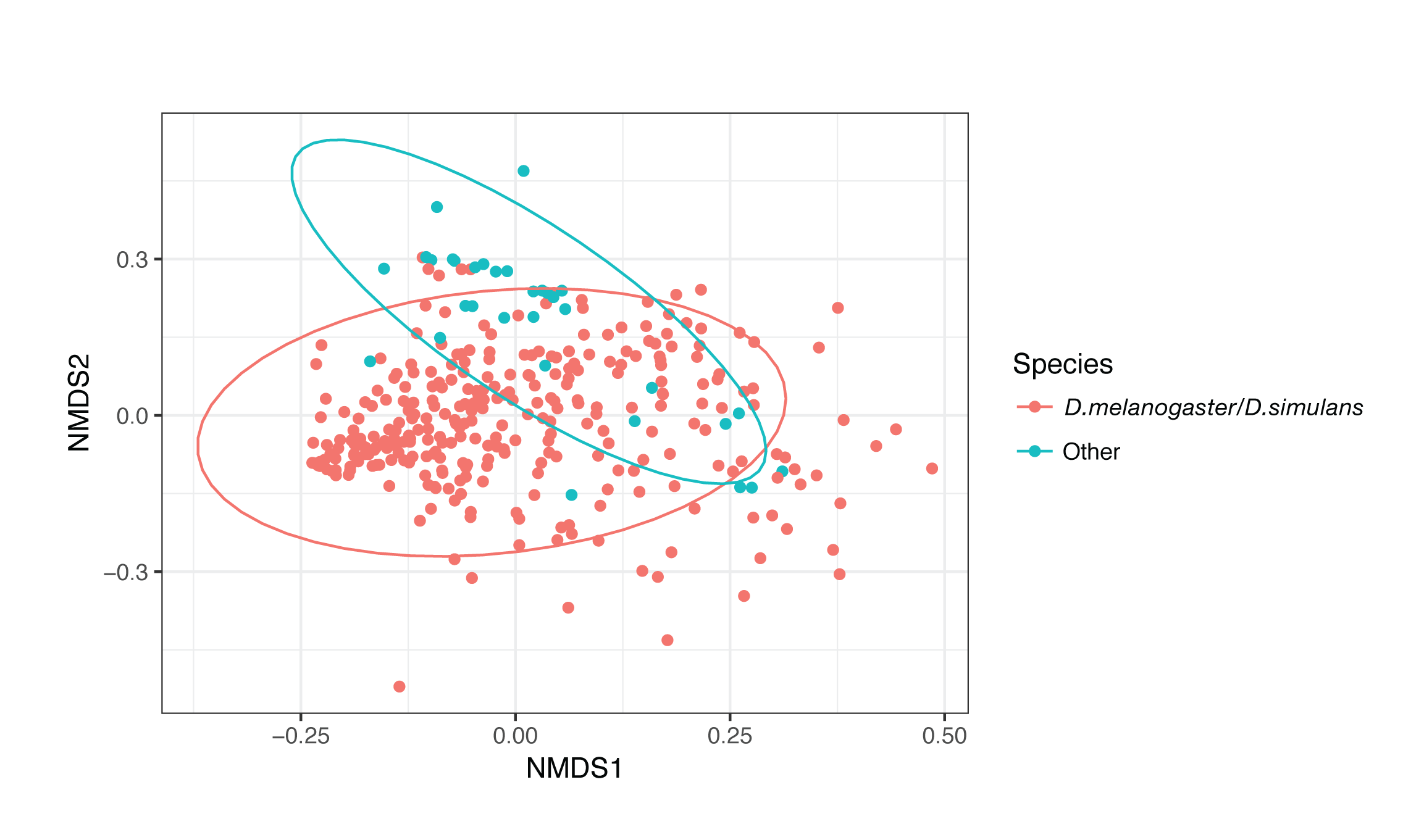

Supplement: S2 Fig — Bray-Curtis dissimilarity NMDS of fungal communities vectored by Drosophila in all vineyards in 2015 and 2016. Each sample was rarefied to 1000 sequences and is represented by a single point, color-coded by species. ADONIS: R2 = 0.018, p = 0.001. (TIF) [file pone.0196440.s002.tif]

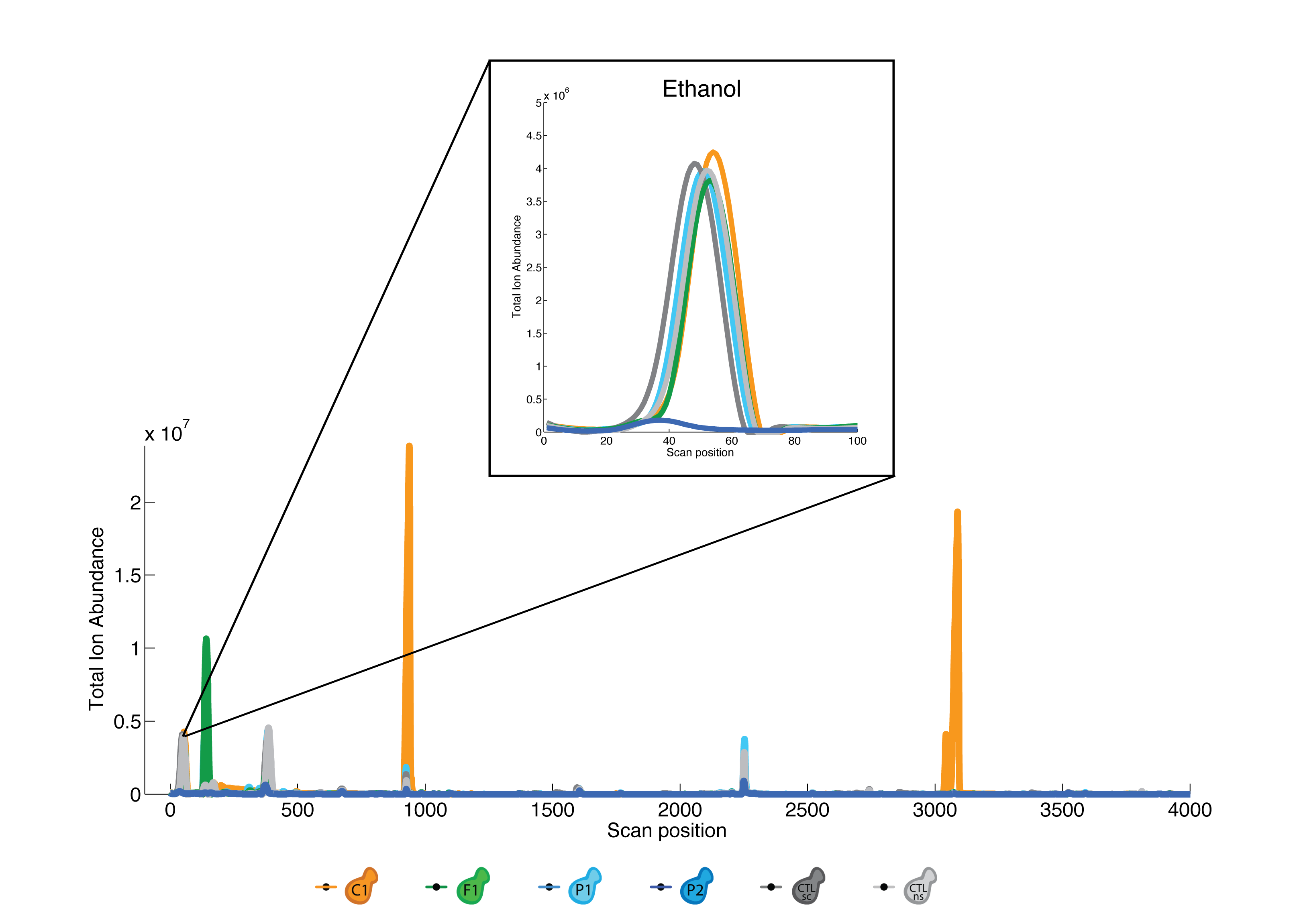

Supplement: S3 Fig — When measured by GC-MS, Pichia manshurica produces very low levels of ethanol (inset) and other volatile metabolites compared to other yeast species on the panel. Each line represents the average of eight GC-MS replicates for a given yeast species. Replicates were sampled for GC-MS in parallel with oviposition assays. (TIF) [file pone.0196440.s003.tif]

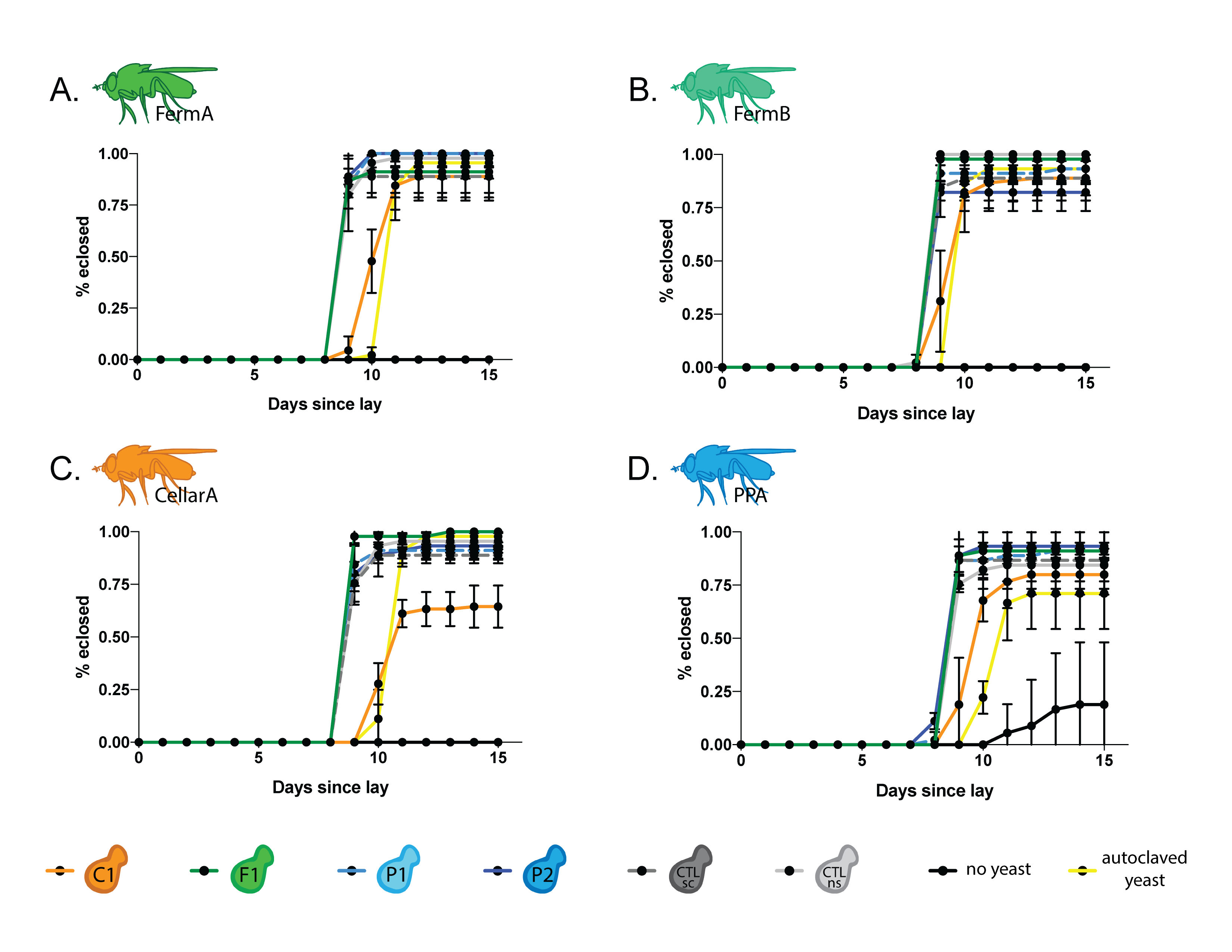

Supplement: S4 Fig — Note that data for larvae that were not given yeast only exist for PPA line because no larvae eclosed without the addition of yeast in any other lines. (TIF) [file pone.0196440.s004.tif]

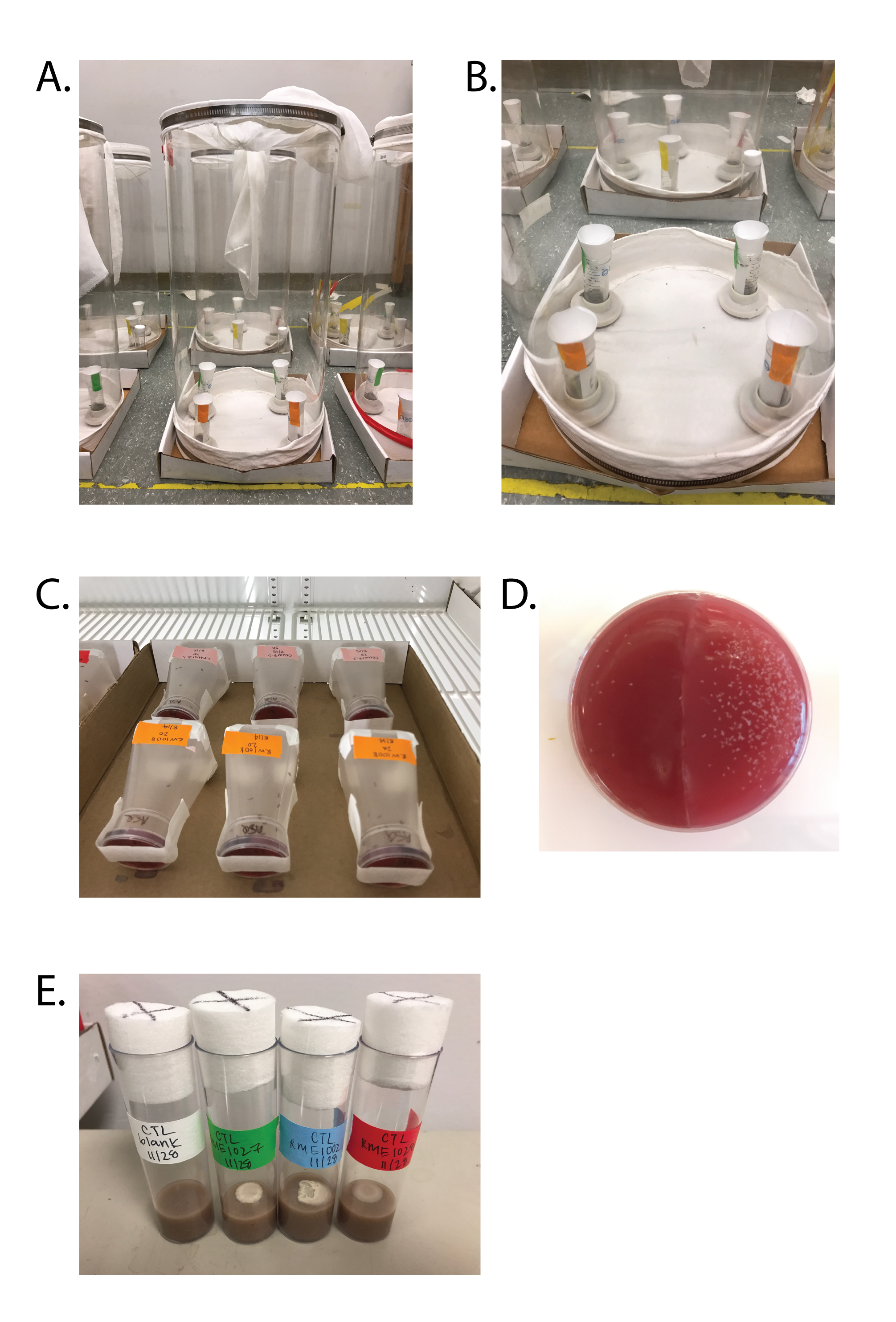

Supplement: S5 Fig — (A) Setup of a single, trap-based olfactory assay. (B) Close up of trap-based olfactory assay. Traps can be arranged in four possible combinations, two of which are depicted here. (C) Setup of six oviposition assays. (D) Example agar plate after oviposition assay. Left side is uninnoculated grape juice agar, right side is yeast inoculated grape juice agar. (E) Larval development and longevity assays were performed in wide vials shown here. Both larvae and adults were exposed to a live monoculture of yeast spotted onto sterile banana media. Depicted are Day 7, negative control vials that had no larvae or adult flies but grew alongside behavioral assays to monitor bacterial or mold contamination. (TIF) [file pone.0196440.s005.tif]

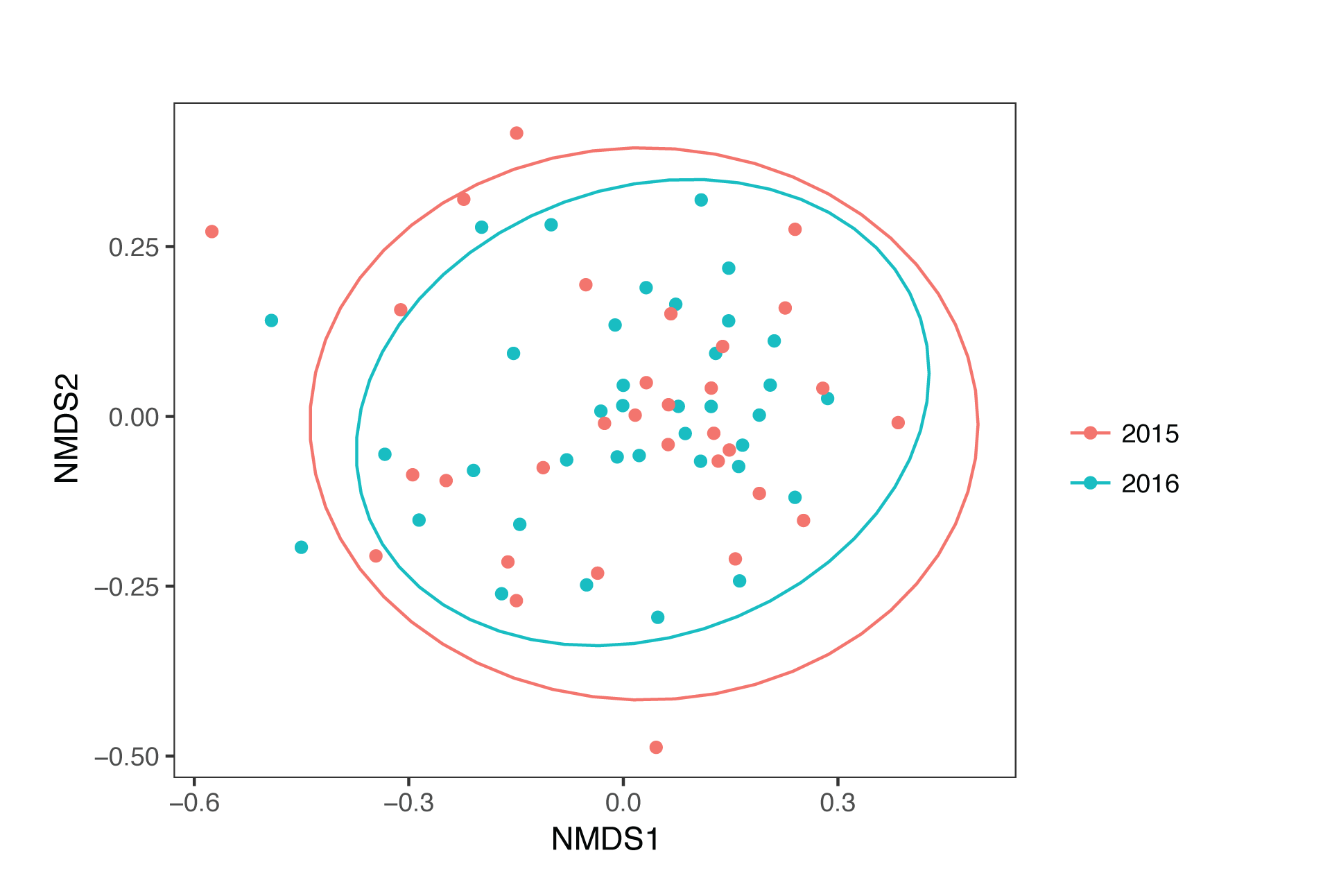

Supplement: S6 Fig — Each sample was rarefied to 200 sequences and is represented by a single point, color-coded by year. (TIF) [file pone.0196440.s006.tif]
