# Supplementary material for: The ecology of the Drosophila-yeast mutualism in wineries
Source: PLoS One. 2018 May 16;13(5):e0196440. doi: 10.1371/journal.pone.0196440 (PMC5955509; doi:10.1371/journal.pone.0196440)
Supplement: S1 Table — (PDF) [file pone.0196440.s007.pdf]

	2015 total reads	2016 total reads	Total OTUs	% original reads retained
**Raw sequences**	8724595	15570284	--	--
**After merging pairs**	8034126	14261774	--	91.77%
**After demultiplexing**	5389671	7311698	--	52.28%
	Combined total reads			
**Removal of resequenced control**	12037090		--	49.55%
**After quality filtering**	11302418		--	46.52%
**After chimera filtering**	11293777		6159	46.49%
**Post OTU picking quality filtering**	7609820		399	31.32%
**Final sequence count**	7609820		399	31.32%
