# Supplementary material for: The ecology of the Drosophila-yeast mutualism in wineries
Source: PLoS One. 2018 May 16;13(5):e0196440. doi: 10.1371/journal.pone.0196440 (PMC5955509; doi:10.1371/journal.pone.0196440)
Supplement: S2 Table — Rarefied to 1000 sequences per sample. (PDF) [file pone.0196440.s008.pdf]

| Vineyard | Year Collected | PCR plate name | mean richness | SE | No. samples |
|---|---|---|---|---|---|
| HLD1 | 2015 | L15 | 31.502 | 9.516 | 84 |
| SCM | 2015 | M15 | 33.253 | 17.22 | 74 |
| HLD1 | 2016 | L16 | 39.771 | 10.537 | 41 |
| HLD1 | 2016 | LF16 | 28.8 | NA | 1 |
| SCM | 2016 | M16 | 33.953 | 7.672 | 47 |
| SCM | 2016 | LF16 | 23.15 | 2.55 | 2 |
| HLD2 | 2016 | D16 | 19.741 | 7.673 | 54 |
| HLD2 | 2016 | LF16 | 25.9 | 5.193 | 10 |
| EBO | 2016 | O16 | 22.027 | 7.209 | 44 |
