# Supplementary material for: The ecology of the Drosophila-yeast mutualism in wineries
Source: PLoS One. 2018 May 16;13(5):e0196440. doi: 10.1371/journal.pone.0196440 (PMC5955509; doi:10.1371/journal.pone.0196440)
Supplement: S3 Table — Each table represents a different yeast species. Within each yeast species, ANOVA was first used to test for any differences in ovipostion responses between fly lines for a given yeast species and denoted by a * next to each yeast isolate. If ANOVA results were statistically significant, Tukey's multiple comparisons test was used to identify the fly lines exhibiting ovipostion responses that were significantly different than other lines and are depicted with * within the table. ns: not significant, *: p<0.05, **: p<0.01, ***: p<0.001, ****: p<0.0001. (PDF) [file pone.0196440.s009.pdf]

| Yeast Isolate: C1** | | | | |
|---|---|---|---|---|
| | FermA | FermB | CellarA | PPA |
| FermA | - | ** | ns | ns |
| FermB | - | - | ns | * |
| CellarA | - | - | - | ns |
| PPA | - | - | - | - |

| Yeast Isolate: F1** | | | | |
|---|---|---|---|---|
| | FermA | FermB | CellarA | PPA |
| FermA | - | * | * | ns |
| FermB | - | - | ns | ns |
| CellarA | - | - | - | ns |
| PPA | - | - | - | - |

| Yeast Isolate: P1**** | | | | |
|---|---|---|---|---|
| | FermA | FermB | CellarA | PPA |
| FermA | - | **** | ns | ns |
| FermB | - | - | *** | ** |
| CellarA | - | - | - | ns |
| PPA | - | - | - | - |

| Yeast Isolate: P2** | | | | |
|---|---|---|---|---|
| | FermA | FermB | CellarA | PPA |
| FermA | - | ns | ** | ns |
| FermB | - | - | ns | ns |
| CellarA | - | - | - | ns |
| PPA | - | - | - | - |

| Yeast Isolate: CTLsc*** | | | | |
|---|---|---|---|---|
| | FermA | FermB | CellarA | PPA |
| FermA | - | *** | ns | ns |
| FermB | - | - | ** | * |
| CellarA | - | - | - | ns |
| PPA | - | - | - | - |

| Yeast Isolate: CTLns**** | | | | |
|---|---|---|---|---|
| | FermA | FermB | CellarA | PPA |
| FermA | - | **** | ns | ns |
| FermB | - | - | *** | *** |
| CellarA | - | - | - | ns |
| PPA | - | - | - | - |
