# Supplementary material for: The ecology of the Drosophila-yeast mutualism in wineries
Source: PLoS One. 2018 May 16;13(5):e0196440. doi: 10.1371/journal.pone.0196440 (PMC5955509; doi:10.1371/journal.pone.0196440)
Supplement: S4 Table — Control conditions are shaded grey. (ANOVA when compared to no yeast control followed by Tukey's multiple comparisons test was used to calculate significance values, ****: p < 0.0001). (PDF) [file pone.0196440.s010.pdf]

		Yeast treatment							
**Fly Line**	**ANOVA**	**C1**	**F1**	**P1**	**P2**	**CTLsc**	**CTLns**	**no yeast**	**Dead yeast, cornmeal media**
**FermA**	****	88.9%	100.0%	100.0%	100.0%	100.0%	97.8%	0.0%	94.4%
**FermB**	****	88.9%	100.0%	93.3%	82.2%	88.9%	100.0%	0.0%	93.3%
**CellarA**	****	64.4%	100.0%	91.1%	93.3%	88.9%	95.6%	0.0%	96.7%
**PPA**	****	80.0%	91.1%	91.1%	93.3%	86.7%	84.4%	20.0%	67.8%
