# Supplementary material for: The ecology of the Drosophila-yeast mutualism in wineries
Source: PLoS One. 2018 May 16;13(5):e0196440. doi: 10.1371/journal.pone.0196440 (PMC5955509; doi:10.1371/journal.pone.0196440)
Supplement: S5 Table — (One way ANOVA followed by Tukey's multiple comparisons test was used to calculate significance values, ****: p<0.0001). (PDF) [file pone.0196440.s011.pdf]

**FermA**	C1	F1	P1	P2	CTLsc	CTLns
C1	-	****	****	****	****	****
F1	-	-	ns	ns	ns	ns
P1	-	-	-	ns	ns	ns
P2	-	-	-	-	ns	ns
CTLsc	-	-	-	-	-	ns
CTLns	-	-	-	-	-	-

**FermB**	C1	F1	P1	P2	CTLsc	CTLns
C1	-	****	****	****	****	****
F1	-	-	ns	ns	ns	ns
P1	-	-	-	ns	ns	ns
P2	-	-	-	-	ns	ns
CTLsc	-	-	-	-	-	ns
CTLns	-	-	-	-	-	-

**CellarA**	C1	F1	P1	P2	CTLsc	CTLns
C1	-	****	****	****	****	****
F1	-	-	ns	ns	ns	ns
P1	-	-	-	ns	ns	ns
P2	-	-	-	-	ns	ns
CTLsc	-	-	-	-	-	ns
CTLns	-	-	-	-	-	-

**PPA**	C1	F1	P1	P2	CTLsc	CTLns
C1	-	****	****	****	****	****
F1	-	-	ns	ns	ns	ns
P1	-	-	-	ns	ns	ns
P2	-	-	-	-	ns	ns
CTLsc	-	-	-	-	-	ns
CTLns	-	-	-	-	-	-
