# Supplementary material for: The ecology of the Drosophila-yeast mutualism in wineries
Source: PLoS One. 2018 May 16;13(5):e0196440. doi: 10.1371/journal.pone.0196440 (PMC5955509; doi:10.1371/journal.pone.0196440)
Supplement: S6 Table — One way ANOVA followed by Tukey's multiple comparisons test was used to calculate significance values, ns: not significant, *: p<0.05, **: p<0.01, ***: p<0.001, ****: p<0.0001. (PDF) [file pone.0196440.s012.pdf]

	FermA	FermB	CellarA	PPA
FermA	-	*	*	ns
FermB	-	-	ns	***
CellarA	-	-	-	**
PPA	-	-	-	-
