# Supplementary material for: The ecology of the Drosophila-yeast mutualism in wineries
Source: PLoS One. 2018 May 16;13(5):e0196440. doi: 10.1371/journal.pone.0196440 (PMC5955509; doi:10.1371/journal.pone.0196440)
Supplement: S7 Table — (PDF) [file pone.0196440.s013.pdf]

Media name	Purpose	Volume	Recipe
**5% YPD, agar**	Solid media for yeast isolation	1L	20g Peptone (BD Bacto Peptone), 10g Yeast Extract (Amresco Yeast Extract, Bacteriological, Ultra Pure Grade), 50g Dextrose (Fisher Scientific Dextrose Anhydrous), 20g Agar (BD Bacto Agar), MilliQ water to 1L. Autoclaved and poured into 100 x 60mm petri dishes (Falcon).
**WLB**	Buffer for DNA extration	-	2M Guanidinium thiocyanate (Fisher Scientific), 0.5 M EDTA (Fisher Scientific), 1.8% Tris base (Promega), 8% NaCl (Sigma Aldrich), 150mL of MilliQ water, and adjust to pH 8.5. Autoclaved and filter sterilized (Nalgene 75mm filter unit, 0.2aPES). (Will Ludington, personal communication).
**Agar grape juice**	Olfactory preference assay	1L	1.7g Yeast nitrogen base without amino acids or ammonium sulfate (Difco, BD), 20g Agar (BD Bacto Agar), 355mL Organic Cascadian Farms Concord grape juice concentrate (1 can), and 645mL MilliQ water. Heated to a boil and poured into 60 x 15mm petri dishes (Falcon).
**Liquid grape juice**	Oviposition assay	1L	(Prepare as instructed on can) 1 can of Organic Cascadian Farms Concord grape juice concentrate, 3 parts MilliQ water. Heated to a boil.
**5% YPD, liquid**	Liquid media for yeast starter cultures	1L	20g Peptone (BD Bacto Peptone), 10g Yeast Extract (Amresco Yeast Extract, Bacteriological, Ultra Pure Grade), 50g Dextrose (Fisher Scientific Dextrose Anhydrous), and MilliQ water to 1L. Autoclaved and filter sterilized (Nalgene 75mm filter unit, 0.2aPES).
**GB media**	Larval development and longevity assay	see recipe	40% weight by volume (w/v) fresh, pureed organic banana, 60% w/v MilliQ water, and 1.25% agar (BD Bacto Agar). Autoclaved and poured into wide mouth *Drosophila* vials (wide mouth, K-resin, Genessee Scientific).
